# A quality by design strategy for cocrystal design based on novel computational and experimental screening strategies: part A

**DOI:** 10.1007/s13346-024-01743-2

**Published:** 2024-11-20

**Authors:** Steven A. Ross, Adam Ward, Patricia Basford, Mark McAllister, Dennis Douroumis

**Affiliations:** 1 Centre for Research Innovation (CRI), University of Greenwich, Medway Campus, Chatham Maritime, Kent, ME4 4TB UK; 2 Department of Pharmacy, School of Applied Sciences, University of Huddersfield, Huddersfield, West Yorkshire HD1 3DH UK; 3 Pfizer Global Research & Development, Ramsgate Road, Sandwich, CT13 9NJ UK

**Keywords:** Cocrystals, Jet dispensing, Molecular modelling, Screening, Quality by design

## Abstract

While pharmaceutical Cocrystals have long been acknowledged as a promising method of enhancing a drugs bioavailability, they have not yet experienced widespread industrial adoption on the same scale as other multi-component drugs, such as salts and amorphous solid dispersions. This is partly due to the lack of a being no definitive screening strategy to identify suitable coformers, with the most cocrystal screening strategies heavily relying on trial and error approaches, or through utilizing a multiple and often conflicting, computational screening techniques combined with high material consumption experimental techniques. From the perspective of industry, this can often lead to high material waste and increased costs, encouraging the prioritization of more traditional bioenhancement techniques. Here we present a strategy for the selection of multicomponent systems involving computational modelling for screening of drug– former pairs based on a combination of molecular complementarity and H-bond propensity screening. Jet dispensing printing technology is co-opted as a mechanism for High-Throughput Screening (HTS) of different stoichiometric ratios, as a low material consumption screening strategy. This strategy is presented herein as a Quality by Design (QbD) crystal engineering approach, combined with experimental screening methods to produce cocrystals of a novel 5-Lipoxygenase (5‐LO) inhibitor, PF-04191834 (PF4). Through this methodology, three new cocrystals were indicated for PF4, confirmed via DSC and XRPD, from less than 50 mg of original testing material. Part B of this study will demonstrate the scalability of this technique continuous extrusion.

**Graphical Abstract:**

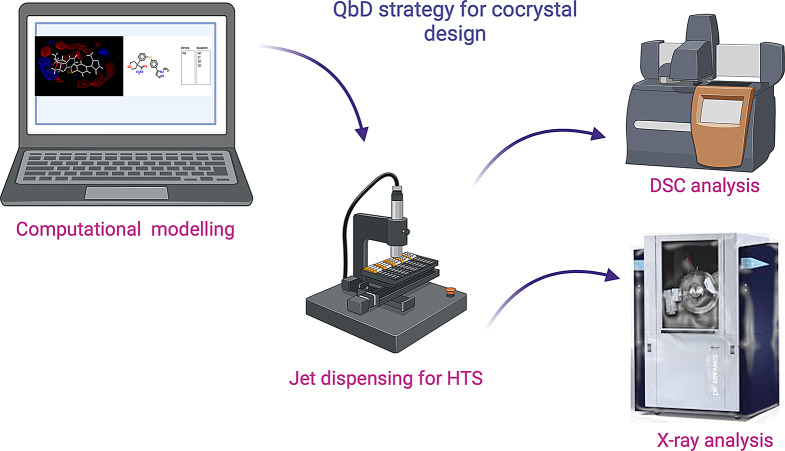

**Supplementary Information:**

The online version contains supplementary material available at 10.1007/s13346-024-01743-2.

## Introduction

Currently, widespread adoption of cocrystallization during drug product development is hindered through difficulties with both screening and manufacture. Until recently, the most common methods of cocrystal formation were based around solvent crystallization methods and mechanochemical milling methods [1]. Methods such as ball-milling are batch processes and have shown to often lack the required energy to fully complete the cocrystallization of the physical mixture [2–5].

Once an API is selected for cocrystallization, it must be screened for potential coformers. However, guidance for the selection of coformers is ambiguous and cocrystal development remains a largely empirical process, hampering industrial utilization of this solid form design option [1]. Energy based screening approaches involve the calculations of lattice energies between the two cocrystal constituents and that of a hypothetical cocrystal structure. Here, the energy differences will be used to assess whether supramolecular hetero or homosynthon formation are more likely [6–8]. While these methods can be effective when screening for known forms, the difficulties lie in the prediction of unknown crystal structures. This involves using anisotropic intermolecular atom-atom potentials, with the electrostatic model and the intramolecular energy penalty for changes in specified torsion angles, derived from ab initio calculations on the isolated molecules [9].

This issue can be avoided through screening in the liquid or gaseous states, circumventing the need for the long-order packing predictions required for solid state lattice energy predictions and simply assuming that all of the solid-state interactions are adequately considered. However, despite the fact these methods are more expedient, they are arguably less precise. In a study of griseofulvin and spironolactone cocrystals from a data-base of 310 potential coformers, the 35 coformers with the highest energy differences were evaluated in experimental LAG screening. Only one cocrystal of griseofulvin and two cocrystals for spironolactone were identified [10]. However, it is not so much the lack of successful cocrystals that makes this screening strategy a tricky proposition, but where those successful coformers were ranked. For both APIs tested, no successful cocrystal appeared in the list of the top 25 coformers ranked according to energy differences, with one coformer actually falling outside of the quoted energy difference cut off point of 11 kJ mol^− 1^ for successful cocrystal formation [11]. This issue was further emphasised by Grecu et al. [12], when screening for new cocrystals of nalidixic acid. Whilst the widely accepted cut-off value for energy difference (< 11 kJ mol^− 1^) from commonly used prediction models certainly does point towards non-formation, there is no clear trend on which to predict the relative likelihood of formation for scores which exceed this value. While screening in the liquid state shows promise, the energy differences are typically small between the cocrystal form and the base components to provide critical insights [7]. Furthermore, there are only a limited number of studies to justify its inclusion.

Alternative screening strategies are based on the likelihood of hetero-hydrogen bond formation and statistical complementarity analysis. Complementarity screening is based on the works of László Fábián, who presented the findings of a large-scale statistical analysis of all current cocrystal pairs in the Mercury CSD to note any molecular motifs that lend themselves to cocrystal formation [13]. In Fábián’s study, 131 molecular descriptors such as bond and group counts, hydrogen bond donor and acceptor counts, size and shape descriptors, surface area descriptors (with partitioned and charge weighted variants), molecular electrostatic descriptors among others were used to characterize the 1949 molecules, without any prior consideration of their importance in cocrystal formation. Molecules found in the same cocrystal were split into pairs and analysed alongside each other, one descriptor per molecule at a time. The study identified 5 key molecular descriptors that showed a much stronger correlation in cocrystals than others. Firstly, it was shown that molecules of similar polarities are far more likely to form cocrystals. The highest correlated descriptor identified from this analysis was the fractional polar volumes (FPV) of the cocrystalized molecules. Another polarity based descriptor which showed a similarly strong correlation was the dipole moment. which is defined as the difference in electronegativity that occurs when there is a separation of charge between two ions in an ionic bond or between atoms in a covalent bond. Cocrystal formers with similar dipole moments were shown to be largely conducive to cocrystallization.

In addition to this, molecules of similar geometries were shown to be much more likely to form cocrystals. During complementarity analysis, the Van der Waals volumes of the molecule are enclosed within a rectangular box, based on the box model of crystal packing [14]. The long, medium and short axis of this box were sorted and whilst on their own they are an indicator of the size of the molecule, their ratios (S/L, M/L and S/M) provide information on molecular shape. It was shown that these axis ratios provide much stronger correlations (between the cell axis length and the molecular dimensions) than the axis lengths, indicating that matching molecular shapes is of greater importance for cocrystal formation than that of absolute molecular dimensions. During complementarity screening, cocrystals with similar S/L, M/L and S/M ratios are preferred for cocrystallization and thus ranked higher.

PF-04191834 (PF4) is a potent, non-redox, selective, competitive inhibitor of the 5-lipoxygenase (5-LOX) enzyme developed for the treatment of mild to moderate asthma [15]. While PF4 is chemically and physically stable, it has an extremely low aqueous solubility with moderate permeability [16]. However, due to its unfavourable biopharmaceutical properties, particle size reduction approaches such as micronization are needed to improve bioperformance from oral formulations and it is therefore hoped that a cocrystal of this API can be identified to address the dissolution and solubility challenges for oral delivery. PF4 was chosen as a model compound to demonstrate the novelty and effectiveness of this screening technique due its physiochemical properties rendering it challenging to bioenhancer through more conventional techniques. It is monomorphic, a weakly basic compound, with the molecule’s pKa rendered it too low for salt formation (pKa basic site 1.81). Furthermore, it has very limited solubility in volatile organic solvents, which has presented difficulties in developing as amorphous solid dispersions.

Here we present a novel strategy for the design of pharmaceutical cocrystals which combines computational and experimental HTS methods. This QbD cocrystal screening approach was developed to save on material and labour costs, making the screening process less resource intensive. HTS is described as a miniaturised, automated process which can rapidly identify active compounds that provide starting points for drug design and an understanding of the interactions important for cocrystal formation [2]. Jet dispensing was chosen over more traditional screening methods such as slurry screening and mechanochemical approaches as these methods are batch controlled, requiring manual operation and in some cases consuming large amounts of material [17].

## Materials and methods

### Materials

PF-04191834 was provided by Pfizer (Sandwich, UK). L-Tyrosine, L-Tryptophan, Hesperitin, Genistein, Biotin, DL-Mandelic Acid, Pamoic acid, Ethylparaben and Thiabendazole were purchased from Tokyo Chemical Industry Co., Ltd (Tokyo, Japan). Pamoic acid and Nicotinamide were purchased from Sigma-Aldrich (Gillingham, Dorset, UK). Sodium dodecyl sulphate was purchased from Sigma-Aldrich (Haverhill, UK). All solvents used for HPLC analysis and Jet dispensing screening were of analytical grade (Fisher Chemical, Loughborough, UK). Graphical abstract was created using Biorender.com.

### Computational cocrystal screening

Ten different conformations of PF4 were generated and these were then screened against a list of 259 Generally Recognised As Safe (GRAS) coformers through 2 cocrystal screening methods. Mercury CSD 4.2 (Cambridge Crystallographic Data Centre, Cambridge, UK) was used to run complementarity analysis of PF4 against the coformer list and to generate the different conformations of PF4. Hydrogen bond propensity analysis was performed using an in-house Python script executed within Windows 10. Initial coformer screening was carried out using CrystalClear™ software (Technobis, Alkmaar, The Netherlands).

#### Coformer generation

Numerous different conformations of the API were generated to perform the complementarity analysis, ensuring the target coformers are flexible and can form cocrystals with a variety of different molecular conformations. If a coformer is only compatible with one conformation, the geometries of the two components are likely incompatible unless under specific processing conditions, unable to be realistically maintained in practice. 9 conformers of the API were generated and screened against the coformer library. Typically, only molecules with 9 N hits were considered, though some exceptions were made in the interest screening variety. Mogul software (Cambridge Crystallographic Data Centre, Cambridge, UK) was used to verify that the conformations generated were realistically possible. Mogul was used to validate the three-dimensional conformation of PF4 by using its vast library of molecular geometry data to show the most likely values the bond length, angle, torsion and ring. Any identified inconsistences within the PF4 crystal structure were edited based on suggested values to be used for restraints during refinement.

#### Complementarity analysis

Using this method, PF4 was screened against different GRAS list coformers for complementarity based upon Lazlo Fábián motifs for ideal cocrystallization. The polarity of PF4 was calculated as a value of dipole moment and N/H fractional amount and screened against these same factors for the coformer library. Cocrystals tend to form between API and coformers of similar polarity, with dipole moment being the strongest correlation. The Van der Waals volume of the molecule is enclosed in a rectangular box, and the long, medium and short axes of this box were calculated. While L, M, and S refer to the size of the molecule, their ratios provide information about molecular shape and it is these ratios that were screened against the same values in the coformer targets. Cocrystallization is more likely with API/coformer pairs of similar geometries, so this method seeks to rank which coformers are the best option based on their geometric similarity to the API.

After this has been completed, coformers are ranked on a pass/fail metric. The minimum pass requirements are denoted in individual molecular complementarity screen data. Coformers that pass all complementarity requirements are considered suitable for cocrystallization. Those that fail are not. Here the coformers are ranked by how many different API conformations pass the complementarity screening. Those that passed more than 50% of conformations were forwarded for hydrogen bond propensity screening.

#### Hydrogen bond propensity screening

The likelihood of all possible interactions for each pairwise system using multi-component hydrogen bond propensity analysis method is based upon the works of Galeks et al. [18]. Propensity prediction is more sophisticated than frequencies of hydrogen bond occurrence as it takes into account explanatory factors like competition, steric hindrance and aromaticity, alongside the chemistry of functional groups, to build a tailored statistical model for the target system. Using this method, the strongest possible homomeric bond (API-API/ Coformer/Coformer) and heterometric bond (API-Coformer) was calculated and the difference subtracted to give a multi component score. A high multicomponent score directly correlates to the increased likely hood that the molecules of the API will form hydrogen bonds with the coformer, as opposed to bonding with molecules of the same type. Hetero hydrogen bonding allows for the formation of supramolecular heterosynthons, the API-coformer pairs that give the highest multi-component scores are therefore more likely to form supramolecular heterosynthons, and thus more likely to cocrystalized.

#### Interaction map generation

An additional tool in Mercury 4.2 allowed for the generation of intermolecular interaction maps from the coformers. These maps present a 3D picture of possible interactions by identifying functional groups, interaction data, steric exclusion, and environmental factors. These are based upon extracted interaction data taken from IsoStar software (Cambridge Crystallographic Data Centre, Cambridge, UK), a knowledge-based library of information of intermolecular interactions derived from both small molecule crystal structures from the CSD and protein-ligand interactions from the protein data bank (Research Collaboratory for Structural Bioinformatics, USA).

#### Coformer ranking

After screens have been carried out, they are ranked in order of complementarity across API molecular conformations, multi-component score, and finally individual complementarity parameters, with similar polarities (H/N molecular fraction and Dipole moment) given importance over geometric factors. The best API-coformer pairs are then further investigated using Mercury software to assess parameters such as hydrogen bond acceptor/donor site fraction, acceptor/donor site accessibility, solubility parameters, miscibility parameters, thermostability, molecular disorder, potential error as well as certain logistical factors (e.g., availability or material in suitable grade) to further assess the suitability of the API-coformer pair.

### Jet dispensing

PF4 was weighed with each coformer to provide a variety of stoichiometric ratios. To provide a solution, these were dissolved in 50 ml of solvent mixtures, in which both API and coformer were soluble. Solvents used in this experiment include Acetonitrile (ACN), Ethanol (EtOH), Pyridine (PYR), and Dimethylsulfoxide (DMSO), either in 1:1 mixtures. The cocrystals were printed using a Dispense Mate583 (583 dispenser Nordson-Asymtek, Maastricht, Netherlands) on a PTFE plate by dispensing four droplets at the time. For the purposes of this study, the nozzle speed and jetting rate were set at 9 mm s^− 1^ and 33 drops s^− 1^, respectively. The fluid pressure used was 10 bar with a nozzle size of 100 μm. The size of the ball tip and the seat was 2.4 mm and 300 μm respectively. The cocrystals were deposited by jetting a solution of the drugs in several parallel lines along the PTFE plate. All printing parameters and droplet placements were set using Fluidmove^®^ for Windows XP (FmXP) software. Droplet dispensing was followed by instant solvent evaporation. After completion of the printing run, the mixtures were left to dry for 10 min before collection for further analysis.

### Differential scanning calorimetry (DSC)

The thermal profiles for the bulk materials, screened cocrystals and HME manufactured cocrystals were examined using a TA Discovery 2500 instrument (TA Instruments, New Castle, USA). The HME extruded cocrystals were also analysed after 9 months storage under accelerated conditions to check for changes in crystallinity. A sample weight of 3.0 ± 0.2 mg was dispensed and placed inside a 40 μm aluminium pan which was then crimped. Each pan was then analysed from 25 to 300 °C at a heating rate of 10 °C/min. Nitrogen was utilized as the purge gas and was supplied at 50 ml/min. All trials were done in triplicate. TRIOS Software (TA Instruments, New Castle, USA) was used for the data integration and evaluation. Universal analysis software (TA Instruments, New Castle, USA) was utilised for displaying numerous thermograms.

### X-ray powder diffraction (XRPD)

XRPD analysis was utilized as a tool to characterize samples were indications of new crystal entities formation is seen in DSC Analysis. This work was conducted using a Bruker AXS D4 Endeavor diffractometer (Karlsruhe, Germany) in theta-theta geometry using the reflection mode. The diffractometer was equipped with a Cu radiation source with a tube voltage and temperature were set to 40 kV and 40 mA respectively. A primary 4o Soller slit was used while the divergence slit was set at 0.6 mm. Diffracted radiation was detected by a PSD-Lynx Eye detector. The data was collected in the Theta-2Theta goniometer at the Cu wavelength from 3.0 to 40.0 degrees 2-Theta using a step size of 0.020 degrees and a step time of 0.3 s. Samples were placed in a silicon low background sample holder and rotated during collection at 15 rpm. All trials were done in triplicate. Data collection was achieved using Bruker DIFFRAC Plus software. EVA phase analysis software (Bruker, Germany) was used to identify peak positions and intensities of the bulk and extruded products.

## Results and discussion

### Theoretical aspects

Complementarity screening on its own is insufficient for accommodating one of the most important factors during cocrystallization, which is hydrogen bond formation. As has been previously described, cocrystal formation is dependent on the formation of supramolecular heterosynthons between two molecules. It is possible for complementary API/coformer pairs to still favour homo-bonding over hetero bonding. In a recent study by Noonan et al. [19], similar methodology was employed, but with the absence of any H-bond propensity screening (HBP). As a consequence, it was found that a number of high-ranking carboxylic acids which displayed good complementarity with the API favoured homodimer formation with like molecules, leaving the large amounts of unreacted API.

**Fig. 1:**
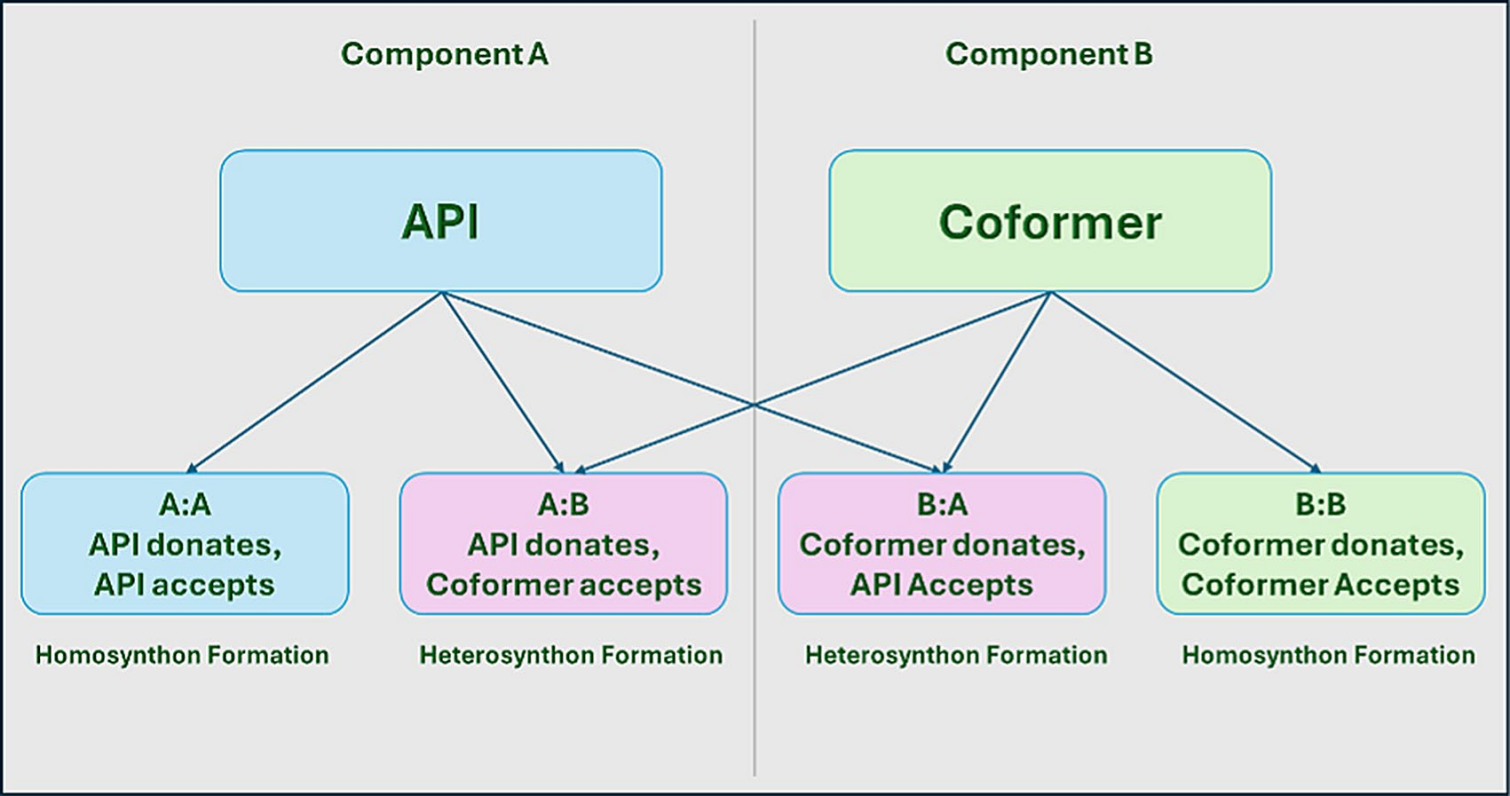
Diagram shows potential hydrogen bonding outcomes during HBP screening

To account for this, HBP screening was performed after the initial complementarity screen, to further rank the coformers. This screening method was co-opted from a 2009 study from Galek et al. [18], who originally employed the methodology for the screening of polymorphs. In the HBP method, potential hydrogen bonds are assigned a propensity, based on their donor and acceptor atoms. For each API-coformer pair, all potential donor-acceptor combinations are identified in which hydrogen bond formation is possible (Fig. 1). For each pair a true/false observation is calculated to decipher whether a hydrogen bond exists or not and a set of molecular/chemical descriptor values are extracted. The existence of every H-bond is determined using geometric criteria: D–A distances less than or equal to the sum of Van der Waals radii + 0.1 A˚, and a D–H/A angle of greater than 120°. All bifurcated (or further subdivided hydrogen bonds) are regarded as two (or more) observations in these studies. The molecular/chemical descriptor information comprises of four key attributes, a competition function, a steric density function (for both the donor and acceptor group), functional group categories (also for both donor and acceptor) and an aromaticity function. These descriptors act as the parameters to which the model functions and can be seen in the scheme below:

1 $$\:\pi=\frac{\text{e}\text{x}\text{p}\:({\alpha}+\sum_{\kappa}\chi{}_{\kappa}{}^{\mathfrak{i}}\:\beta{\kappa})}{1+\text{e}\text{x}\text{p}\:({\alpha}+\sum_{\kappa}\chi{}_{\kappa}{}^{\mathfrak{i}}\:\beta{\kappa})}$$

Formula function for the standard probability distribution used to describe binary data of hydrogen bond propensity calculation. $\:{\alpha}$ is the intercept or baseline variable, and the *βκ* coefficients represent the influence on the propensity to hydrogen-bond, *π*, of their corresponding parameter, $\:{\chi}_{\kappa}^{\mathfrak{i}}$. The α and the $\:{\beta}_{\kappa}$ set are optimized to best reproduce all true and false training observations using a logistic regression procedure [20].

The functionality of this screening method was demonstrated by Delori et al. [21] who used HPB screening to rank the probabilities of hydrogen bond formations during cocrystallization experiments for the drug pyrimethamine. Using this screening method, it was possible to screen multiple coformers and rank, not only based on hydrogen bond propensity, but also what type of interaction (hetero or homo) was more likely to take place. By screening in this manner, one is able to compare the propensity of either the API or coformer forming hydrogen bonds with like molecules as opposed to with each other. By subtracting the highest propensity homo interaction from the maximum hetero interaction, one can obtain a “multi-component score” which represents the likelihood of how the bonds will form. This information can be very useful for the design of a cocrystal screening experiment. By reducing the number of coformers to those most likely to result in successful cocrystal both time and money can be saved, and the efficiency of crystallization can be increased.

### Computational screening results

PF4 was screened against 159 potential GRAS list coformers using Mercury software 4.2 for potential cocrystallization. The coformers were screened against 9 different generated conformations of PF4. This was to ensure that the coformers were complimentary to the API regardless of conformation, increasing the likelihood they could be isolated through laboratory experimentation. The coformers were initially ranked by their closeness (optimal deviancy) to the API. They were then further screened via hydrogen bond propensity to ensure they are likely to form supramolecular heterosynthons. Once a ranking order has been established, the top 25 cocrystals were closely examined using Isostar software to generate interaction maps, identify possible interlinkage through functional groups, check for steric exclusion, identify disorder and possible suspect CSD data to give the list the highest possible reliability.

To ensure the structures generated here were realistic, a Mogul geometry check was performed to analyse the bond lengths, valance angle, torsion angle and rings to check for values outside the expected ranges. The bond lengths, valance angle torsion angle and rings of the base PF4 structure and the conformations can be found in Table S1 (Supplementary Materials).

The geometric values were measured in their M/L axis ratio Δ, S/L axis ratio Δ and the S axis (Å) Δ. These values measure how close in shape the coformers are to PF4. The cut off points for these measurements can be seen in Table 1. If the difference ratios of the coformers fall within these values, they are estimated to be close enough to that of the API to be deemed complementary. The dipole moment magnitude is expressed using the unit Debye (D), which is equal to 3.336 × 10^− 30^ C and for the base PF4 form is 6.434 D. Any coformer with a dipole moment within 5.94 D to the PF4 conformation it is screened against is deemed to be complementary. The last value being observed here is the fraction of polar atoms N, O and S in the molecule which is expressed as FPV. Any coformer with an FPV delta of less than 0.294 FPVΔ is deemed to be complementary to PF4. All these values are slightly different across the different conformations and thus the complementarity of each coformer will vary across conformations. Only coformers which fell within these parameters were deemed to have passed the complementarity screening [13].

**Table 1:**
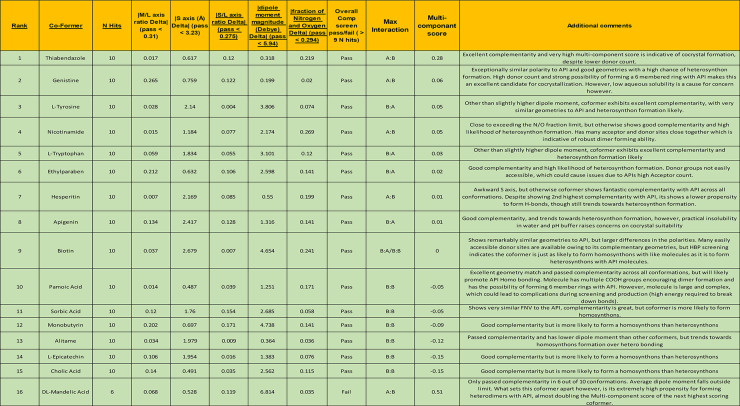
Table summarizing refined computational coformer screening rankings

The complementarity screening works on a Pass/Fail metric, but an “Optimal deviance” was calculated from the complementarity difference metrics to help rank the coformers based on their complementarities. The optimal deviancy describes the percentage closeness of the coformers molecular descriptors to that of the APIs, as guided by Fábián rules for complementarity screening [13] (only conformations for which the coformers passed the complementarity screening were taken into consideration). This provides an arbitrary ranking system for the bulk list, to help simplify the data sets within the main screening list. Using this metric, the higher the percentage, the closer individual molecular descriptors are to exceeding the defined pass/fail limits. Whilst this metric is not wholly accurate as it does not account for cases where one coformer descriptor could be very close to that of the API, while another is close to exceeding the limits, it provides a general view on what coformers are more likely to be complementary with the API. The coformers were ranked by how closely their key descriptors were to that of the API, and for how many conformations they passed the screening. This allowed for informed decision making on what coformers to dedicate further screening and evaluation.

**Fig. 2:**
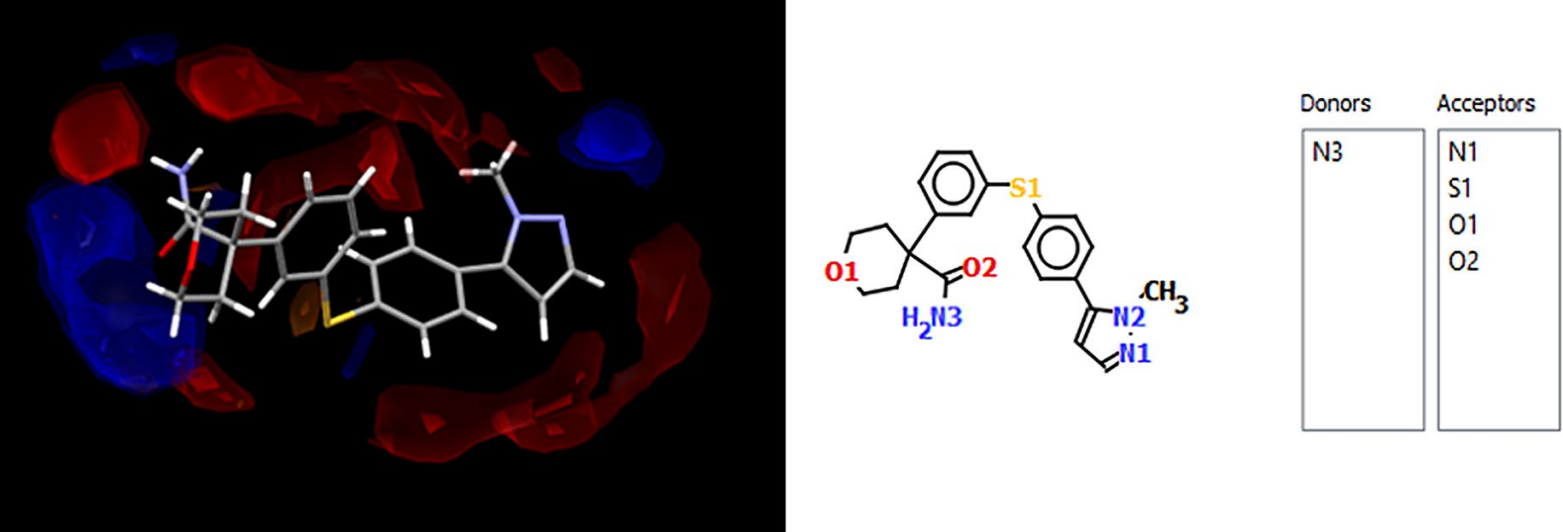
Full interaction map of PF4 showing all interaction points for Donors (Blue) and Acceptors (Red)

The HBP screening helped to further solidify these rankings by predicting the most likely hydrogen bonding outcome (Fig. 2) and the maximum interaction available. Coformers were then further ranked based on their multi-component score (maximum homomeric interaction subtracted by the maximum heterometric interaction). This provides insight into which coformers are most likely to form supramolecular heterosynthons with the API. As HBP screening requires significant processing power, this screening was limited to the top 60 coformers in the interest of saving computational time. In theory, any coformer ranked below this point would have failed their complementarity screening on more than half of the conformations available, and thus would be ineligible for selection anyway.

At this stage, the list had been narrowed to a few key candidates which showed both good molecular complementarity and a propensity towards heterosynthon formation. For these coformers deeper investigation followed to further refine the rankings. This included the generation of an interaction map to further investigate the functional groups contained within these coformers. This allowed for the possibility to identify potential trends towards interaction utilizing IsoStar software (Cambridge Crystallographic Data Centre, Cambridge, UK). The approach also provided the opportunity to disqualify certain coformers based upon molecular disorder, suspect crystal structure data (a number of structural data submitted to the CSD sometimes leave out space group information, lending doubt to their complementary values legitimacy), similarity to other coformers (in the interests of maintaining a varied screening set, similar molecules were excluded) and duplicates. In a number of cases, coformers were also excluded based upon their cost per gram rendering them uneconomical and thus unsuitable for continuous manufacture. The ranking list from Table 2S (Supplementary Materials) was inspected and the top 16 were chosen based on their max interaction, similarity with PF4s polar and geometrical motifs, and their available interaction points and complementary functional groups. The results of this refined list can be seen in Table 1.

Thiabendazole (THI) was highlighted as giving the best possible chance of forming a cocrystal with PF4. After the initial complementarity screening, it was shown to have the lowest optimal deviancy from the API, indicating it is the most complimentary molecule of the entire screen list. Upon further HBP screening, THI was also shown to be most likely to accept donors from PF4, giving a max interaction of 0.28.

**Fig. 3:**
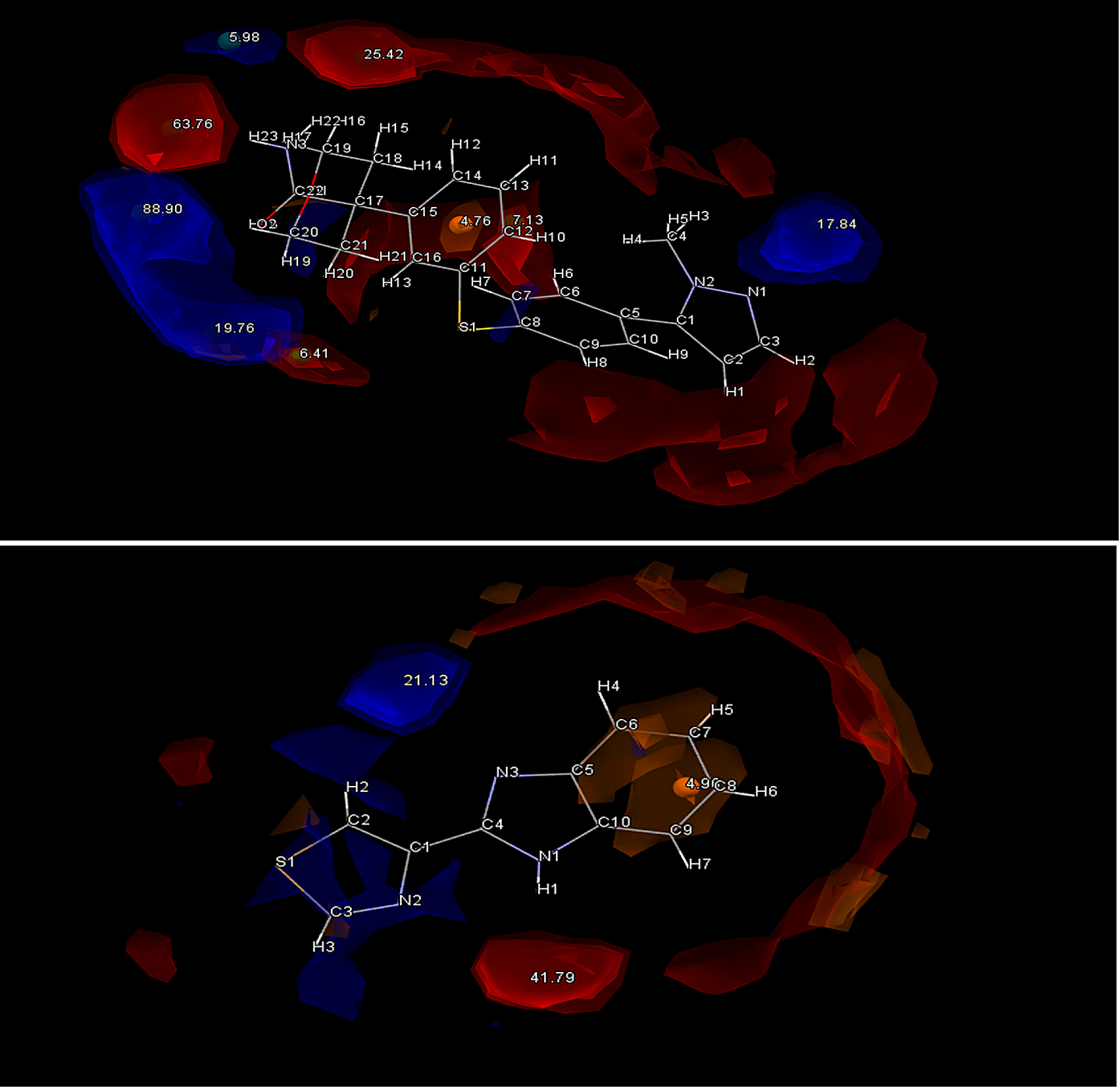
Full interaction maps generated for PF4 (Top) and THI (Bottom). Red spaces indicate Acceptor groups and Blue spaces indicate Donor groups. The highest point of interaction is the N-H amino group on THI and the O_2_ from the carbonyl group on PH_4_

Another factor that gave a strong indication supporting successful cocrystallization was revealed after generating interaction maps for PF4 and THI using Mercury software. As seen in Fig. 3, the most likely point of interaction can be seen in the donor O_2_ part of the carbonyl group in PF4, while the most likely point of interaction for THI can be seen at the N1-H1 acceptor. Previous studies have shown a trend of strong hydrogen bond formation between carbonyl groups and N-H acceptor groups establishing robust synthons described as the N − H(amino)···O(carbonyl) [22].

The max interaction predicted in the HBP screening is that PF4 would donate to THI, so given that these functional groups are also the highest energy interaction hotspots, it seems likely that a robust supramolecular heterosynthon could form between these points, forming a cocrystal. When considered alongside the complementary polarity and geometry data, THI appears to be the most likely candidate for cocrystallization.

**Fig. 4:**
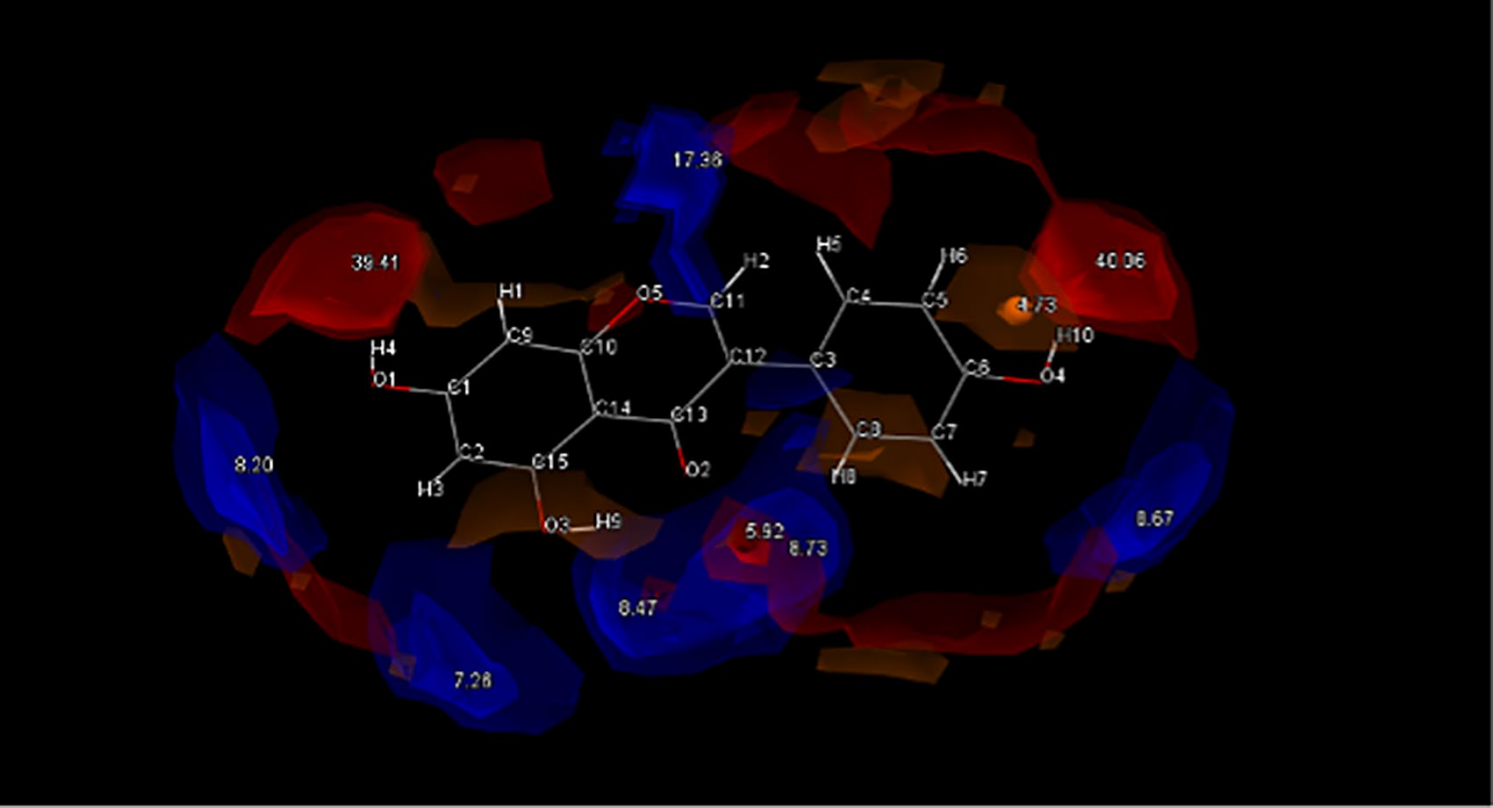
Full interaction maps generated for GEN. Red spaces indicate Acceptor groups and Blue spaces indicate Donor groups. The highest point of interaction is O_1_H_4_ and O_4_H_10_ hydroxyl groups on either end of the molecule

Similarly, high confidence was identified for Genistein (GEN), due to its extremely complimentary polarities with PF4 as well as sharing complimentary geometries. As seen in Fig. 4, the most likely points of interaction are found at hydroxyl groups O_1_H_4_ and O_4_H_10_ of GEN. These points are highly likely to accept a donated N to form a OH⋯N hydrogen bond, which can lead to supramolecular heterosynthon formation.

This was previously shown in a study on Daidzein cocrystals, where it was found that coformers with carbonyl groups were likely to form synthons with the free hydroxyl groups OH molecules on three different coformers [23]. Here, numerous NH2…. OH bonds were seen between the hydroxyl (OH) and carbonyl (C = O) groups, with 7 possible supramolecular synthons predicted. This suggests that a PF4-GEN paring is favourable.

**Fig. 5:**
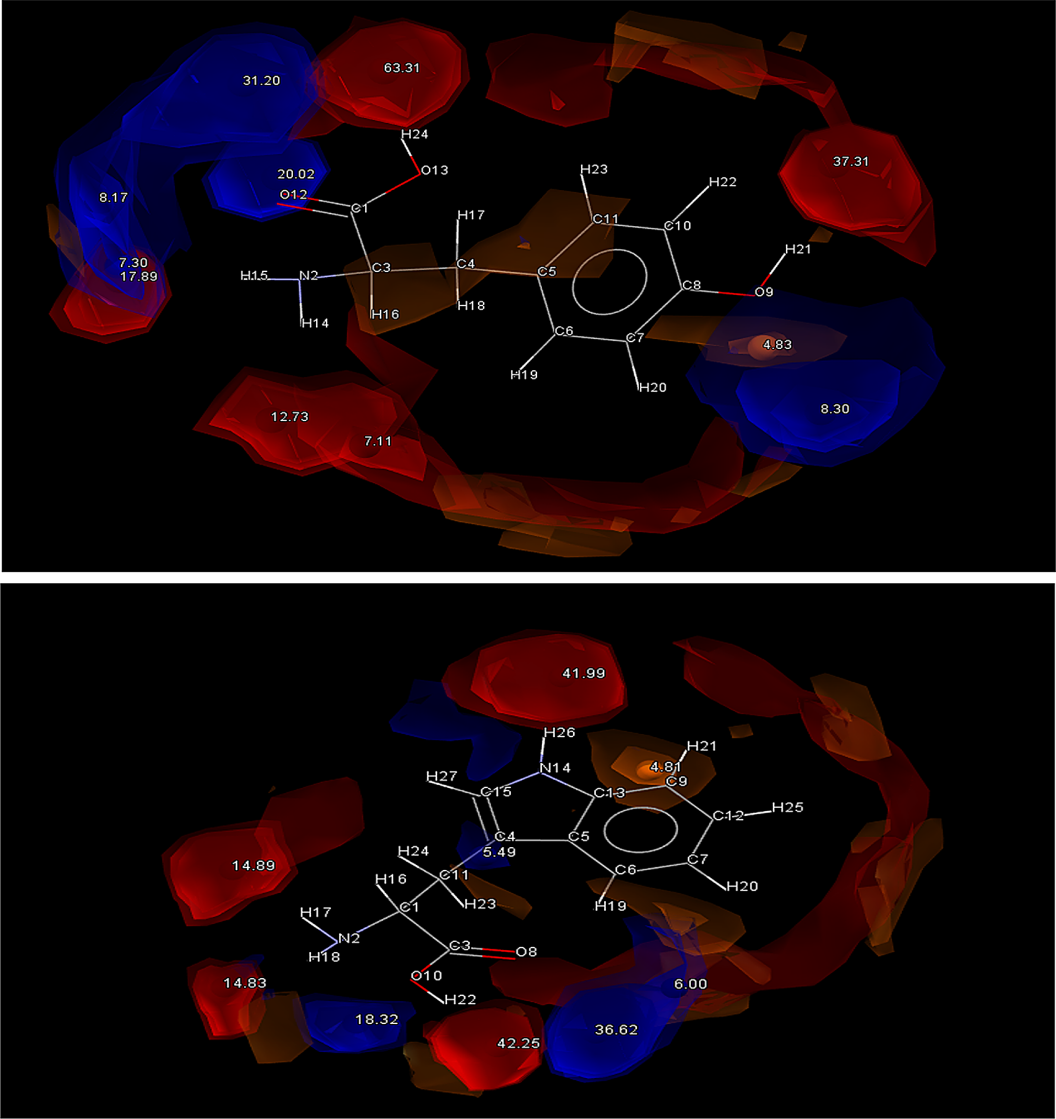
Full interaction maps generated for LTY (Top) and LTP (Bottom). Red spaces indicate Acceptor groups and Blue spaces indicate Donor groups. The highest point of interaction is the HO_13_-O_12_ group from LTY and the O_10_H-O_8_ group from LTP

The HBP screening predicts PF4 will donate to form a synthon, with the N3 being the most likely option, potentially forming a H bond with the highly charged O1H4 and O4H10 acceptors found on either end of the molecule. L-Tyrosine (LTY), L-tryptophan (LTP), Biotin (BIO) and Pamoic acid (PAM) were all considered good options due to the strong correlations between COOH ··· N_arom_ supramolecular heterosynthons forming between coformers containing alcohol and hydroxyl groups and API with an aromatic nitrogen [24, 25]. This pairs well for LTY and LTP, which both contain COOH and OH groups. This trend is supported by the interaction hotspots of LTY and LTP (Fig. 5) which indicate the COOH groups on both molecules have the highest interaction potential. It is hoped a H-bond can form here between the donating O motifs from LTY and LTPs COOH groups and the aromatic N3 from PF4. Both LTY and LTP gave positive indications of heterosynthon formation after HBP screening, with the coformers donating being the most likely combination. In this case LTY and LTP were predicted to form a hydrogen bond with the aromatic N1 acceptor found on PF4. LTY ranked 4th and LTP ranked 7th on the initial complementarity screen, but both were selected to move higher in the final list due to their high propensity for hydrogen bonding. Based on this both coformers were selected for experimental trials.

**Fig. 6:**
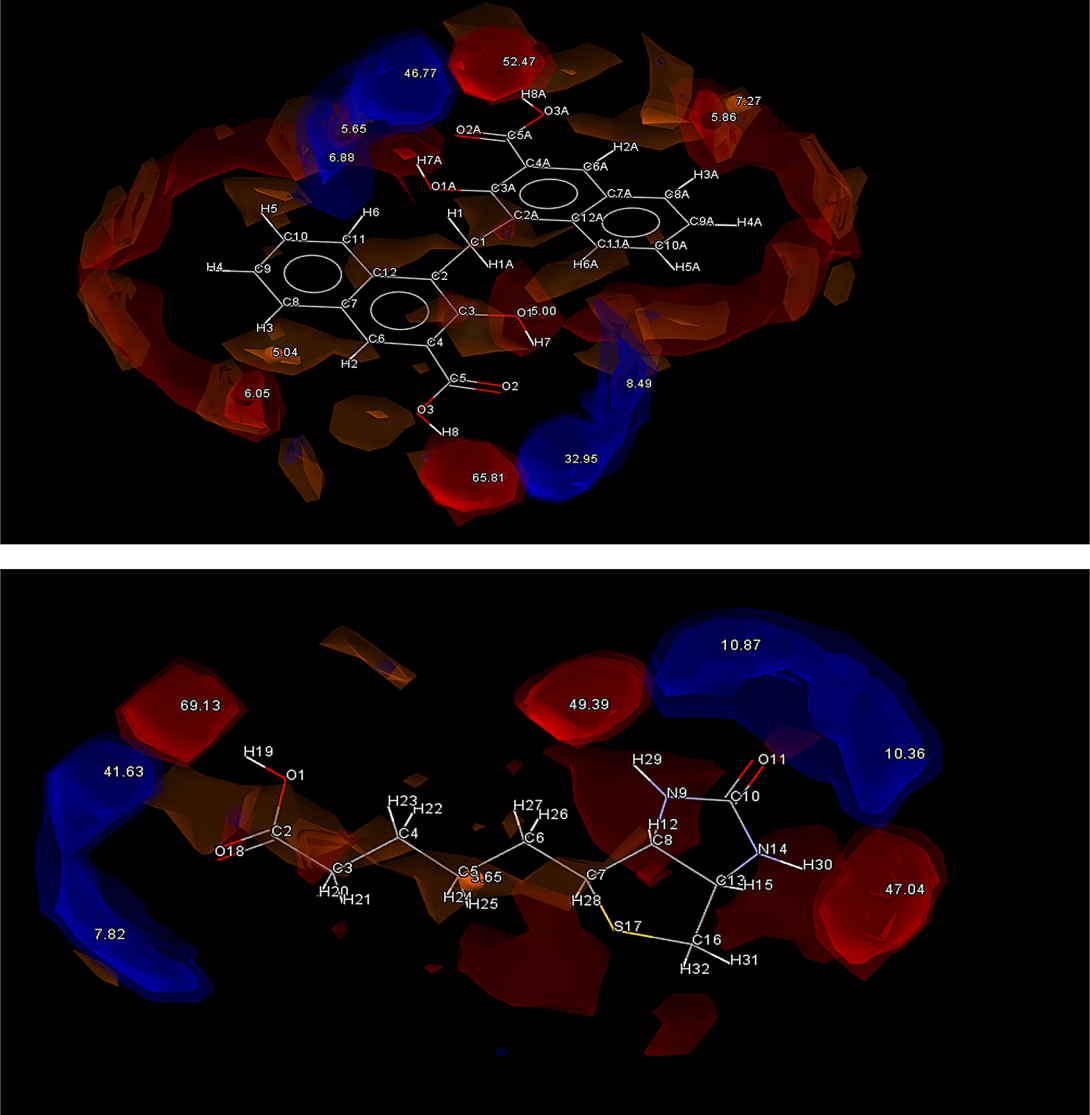
Full interaction maps generated for PAM (Top) and BIO (Bottom). Red spaces indicate Acceptor groups and Blue spaces indicate Donor groups. The COOH groups on both molecules are predicted to be the most likely interaction points

PAM was a slightly different case; despite displaying excellent complementary qualities in regard to both shape and polarities, and containing multiple COOH and aromatic OH groups, the molecule was predicted to prefer homosynthon formation. This could potentially be attributed to the complex geometries of pamoic acid and trend for molecules with multiple COOH groups to form homosynthons with like molecules [25]. Generating the interaction maps from PAM confirmed the likelihood of interaction from the two COOH groups (Fig. 6). Nevertheless, PAM was chosen as one of the 10 coformers to advance to experimental screening due to its strong complementarity results. It was the only coformer forwarded for experimental screening that returned a negative HBP result. A unique coformer among the field was BIO which demonstrated an identical likelihood of forming heterosynthons as it is homosynthons. Despite this BIO was forwarded for experimental screening due to its exceptionally complementary shape, with the hope that an easily accessible and highly charged COOH group would allow the formation of a COOH ··· N_arom_ heterosynthon.

**Fig. 7:**
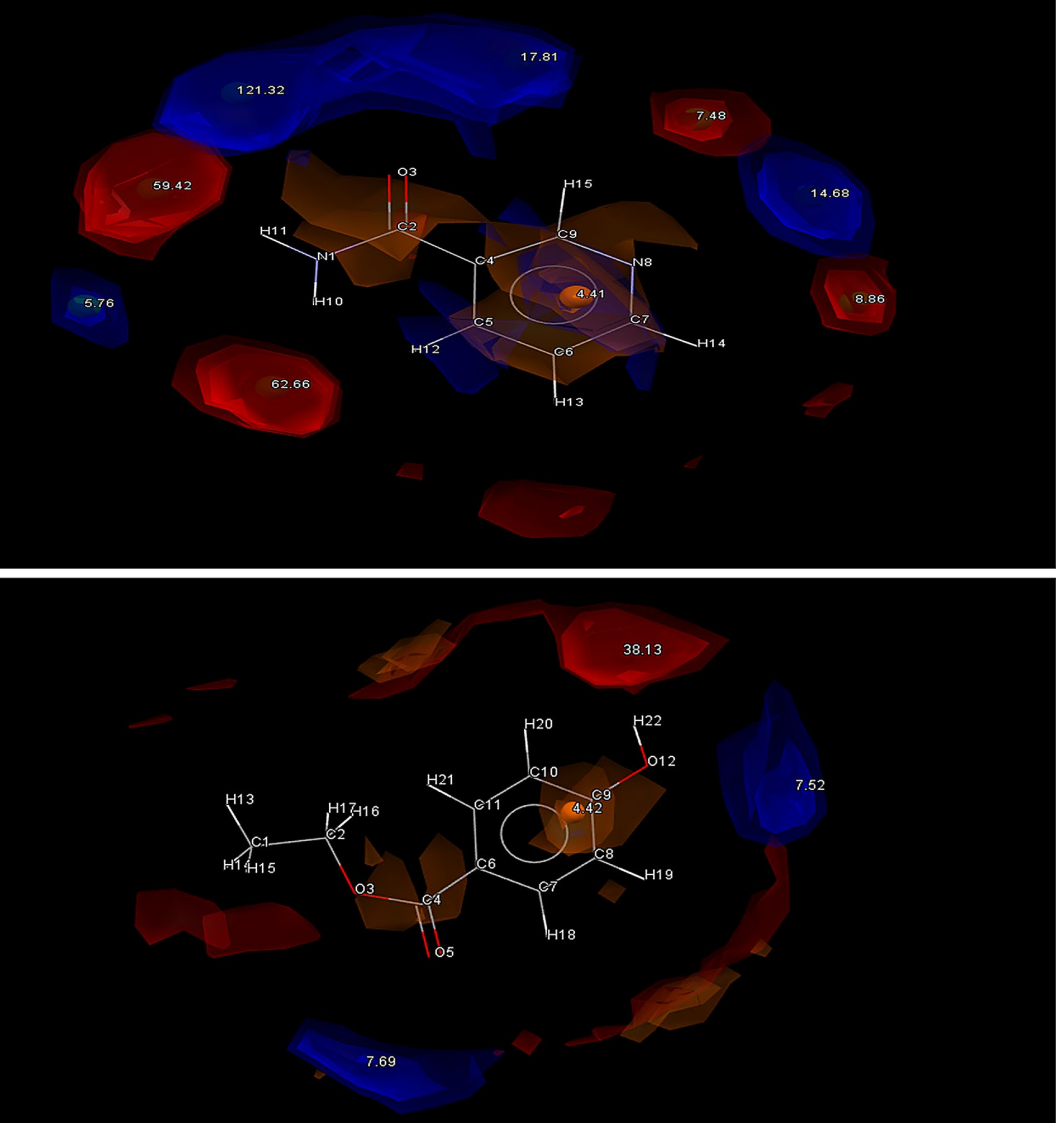
Full interaction maps generated for NIC (Top) and ETB (Bottom). Red spaces indicate Acceptor groups and Blue spaces indicate Donor groups

Nicotinamide (NIC) shows promise as a coformer and thus was selected as a candidate for experimental screening. It only ranked at 22 in the complementarity screen, mostly owing to its high N/O fractional volume, but it showed a high propensity for hetero-hydrogen bonding. Both PF4 and NIC have aromatic N atoms as well as carbamoyl groups, which gives good reason to believe robust dimers may form between these two molecules [26]. Previous studies between nicotinamide and other amides have demonstrated their capacity to form strong centrosymmetric dimers with carbamoyl groups (N − H(amide)···O(carbonyl)) and pyridyl groups (N − H(amide)···N(pyridyl)), both of which are present in PF4 [22, 27]. NIC showed numerous strong donor and acceptor atoms making it an ideal candidate (Fig. 7).

Ethylparaben (ETB), ranked 8th on the initial complementarity screen and shows a tendency to form heterosynthons, with ETB donating to PF4, so on that basis was advanced to experimental screening. However, upon further investigation of the molecule’s interaction points, the hydroxyl group donor atoms display a relatively low chance of interacting. Despite this, OH hydroxyl groups historically show a strong predisposition to forming heterosynthons with amine NH_2_ groups, so it is hoped a NH2…. OH H-bond would form between ETBs O5 donor atom and PF4s N3H23 acceptor group [23, 28].

**Fig. 8:**
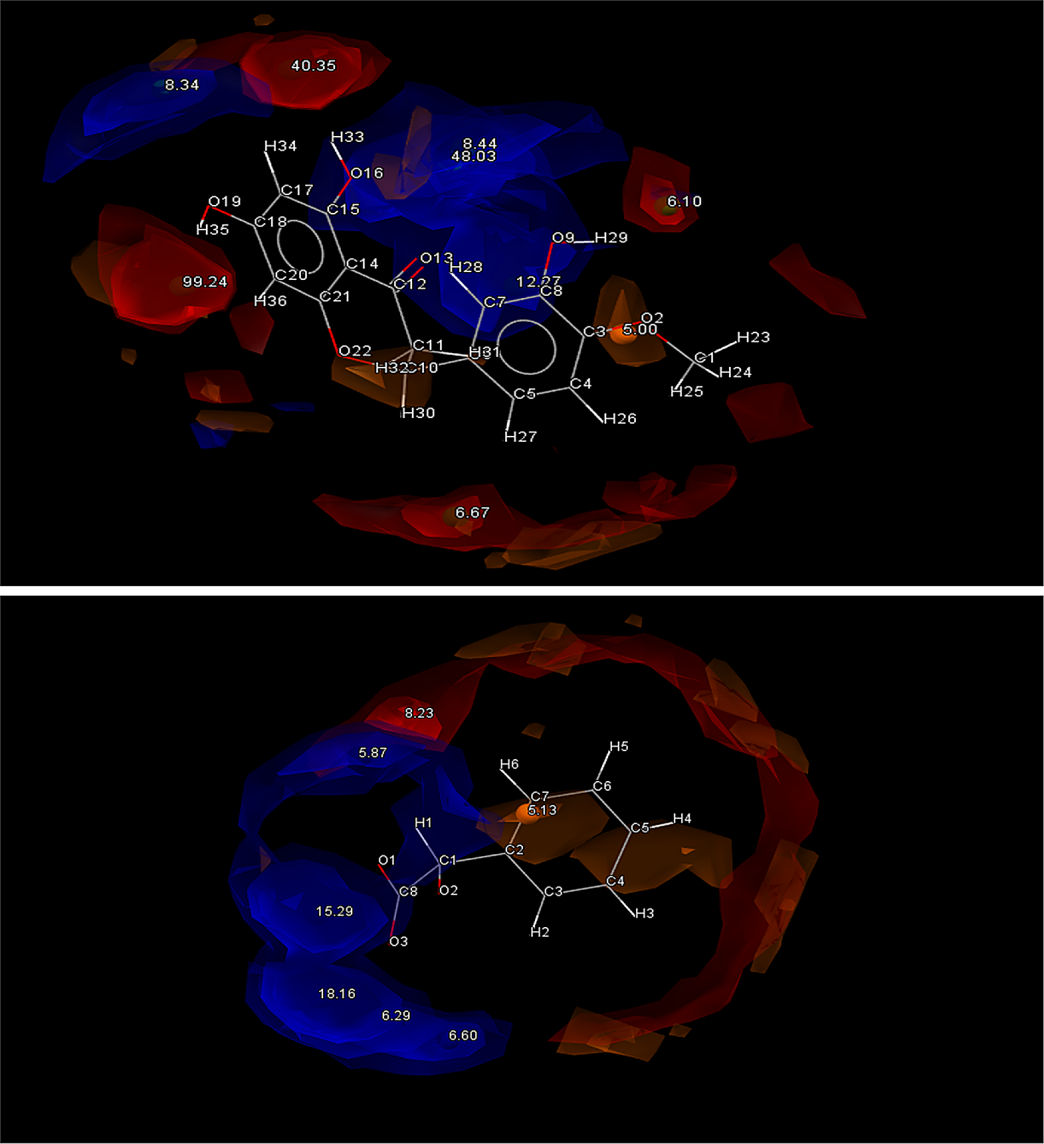
Full interaction maps generated for HES (Top) and DLM (Bottom). Red spaces indicate Acceptor groups and Blue spaces indicate Donor groups

Hesperitin (HES) was seen to be a promising candidate for cocrystallization due to it being ranked 2nd overall on the initial complementarity screen, showing great similarities in shape and polarity to PF4, as well as possessing a large number of acceptor OH groups. This allows for many different options for a donated N from PF4 to form a intermolecular H bond with, indicating the formation of a OH ··· N_arom_ supramolecular heterosynthon is likely (Fig. 8). A less likely candidate in DL-Mandelic acid (DLM) was also forwarded to experimental screening, owing to its high predicted propensity for H-bond formation with PF4. Its multi-component score of 0.51 far exceeded any other coformer tested. However, it only passed the complementarity screening in 6 out of 10 generated conformations, with the dipole moment falling out of the acceptable range in the other 4. Despite this difference in polarity, DLM was still chosen, to evaluate if the larger propensity for H-bonding formation will overcome this shortcoming.

### Experimental screening results

The best performing cocrystals from the computational screening were advanced to experimental screening with jet dispensing utilized for cocrystal formation. The technology utilizes a pneumatic piston with a ball– tip that pushes the solvent solution (drug– coformer) at the jet nozzle tip. Jet dispensing can print rapidly 100 drops/s with volumes as small as 1 nL. It must be stressed, that despite favourable data for complementary and H-bond formation for a given API coformer pair, there is still every chance the system could fail to form cocrystals in practice [1, 29]. These screening methods simply narrow down the coformer list based on trends, as opposed to solid rules. Therefore, experimental validation is essential to confirm cocrystal formation at a small-scale before scaling up to continuous manufacture. Keeping in line with QbD screening methodology, these methods should be rapid, automated and provide enough evidence to further refine the coformer selection process. Jet dispensing technology was selected as it qualifies as a HTS method with low material consumption, vastly reducing the cost and time spent experimentally screening materials compared to more conventional methods such as ball milling or solvent evaporation [17, 29]. The jet dispensing process also allows for rapid screening of different stoichiometric ratios, to find the ideal ratio to synthesize a stable cocrystal form. The ratios in this investigation were 1:1, 1:1.1, 2:1 and 1:2 as studies have shown that they would give a good indication as to whether cocrystallization is possible in either equal ratios, or if a greater amount of either the API or coformer would tip the balance towards creating a stable structure during DSC analysis [30, 31]. A recently published study has found that a stoichiometric ratio of 1:1.1 led to the creation of a β-sitosterol/ 4-hydroxybenzoic acid cocrystal solid solutions in situations where the standard 1:1 did not [32]. For this reason, a 1:1.1 ratio was also investigated as part of the laboratory screen. The solvent media, in which the PF4 and coformer were dissolved in prior to jet dispensing, were selected based upon the shared solubility of the mixture in said solvent.

DSC was selected to analyse the resulting materials, due to its small sample requirements, quick turnover in results and long-established use for cocrystal characterization [22–27, 33]. The purpose of this experimental screening was to indicate whether cocrystal formation will be possible during an HME process. Single endothermic peaks, with no evidence of API or coformer residue was the desired result that would qualify an API/coformer pair for further study. Indications of impure cocrystals (i.e., instances where a results indicate crystallization has taken place, but traces of the parent compound remain in the mixture), such as a broad melting endotherm or gradual return to baseline were ignored in this instance. This was decided upon due to the expectation that a pure cocrystal can be produced in the more energy intensive environment of the HME process. The results of the experimental screening can be seen in Table 2.

**Table 2:** Table showing the potential cocrystallization of PF4 with 10 different coformers, in different stoichiometric ratios, in relevant solvent media, screened through jet dispensing

	Coformer	Max interaction	Multi-component score	Opt dev	Solvent	Active-coformer ratio	Potential cocrystal
F1	Thiabendazole	Active → Coformer	0.28	25.7	ACN: EtOH	1:1	✓
F2						1:1.1	✓
F3						2:1	✗
F4						1:2	✗
F5	Biotin	Active → Coformer Active ← Coformer	0.12	40.1	EtOH: DMSO	1:1	✗
F6						1:1.1	✗
F7						2:1	✗
F8						1:2	✗
F9	Genistine	Active → Coformer	0.06	33.6	ACN: EtOH	1:1	✓
F10						1:1.1	✓
F11						2:1	✓
F12						1:2	✓
F13	L-Tyrosine	Active ← Coformer	0.05	32.9	ACN: DMOS	1:1	✗
F14						1:1.1	✗
F15						2:1	✗
F16						1:2	✗
F17	Nicotinamide	Active → Coformer	0.05	45.5	EtOH: DMSO	1:1	✗
F18						1:1.1	✗
F19						2:1	✗
F20						1:2	✗
F21	L-Tryptophan	Active ← Coformer	0.03	33.8	ACN: DMSO	1:1	✗
F22						1:1.1	✗
F23						2:1	✗
F24						1:2	✗
F25	Ethylparaben	Active ← Coformer	0.02	34.9	EtOH: DMSO	1:1	✗
F26						1:1.1	✗
F27						2:1	✗
F28						1:2	✗
F29	Hesperitin	Active → Coformer	0.01	31	ACN: EtOH	1:1	✓
F30						1:1.1	✓
F31						2:1	✗
F32						1:2	✗
F33	Pamoic Acid	Coformer → Coformer	-0.05	35.9	PYR: DMSO	1:1	✗
F34						1:1.1	✗
F35						2:1	✗
F36						1:2	✗
F40	DL-Mandelic Acid	Active → Coformer	0.51	41.1	EtoH: DMSO	1:1	✗
F41	Nicotinamide	Active → Coformer	0.05	45.5	EtOH: DMSO	3:1	✗
F42						4:1	✗

**Fig. 9:**
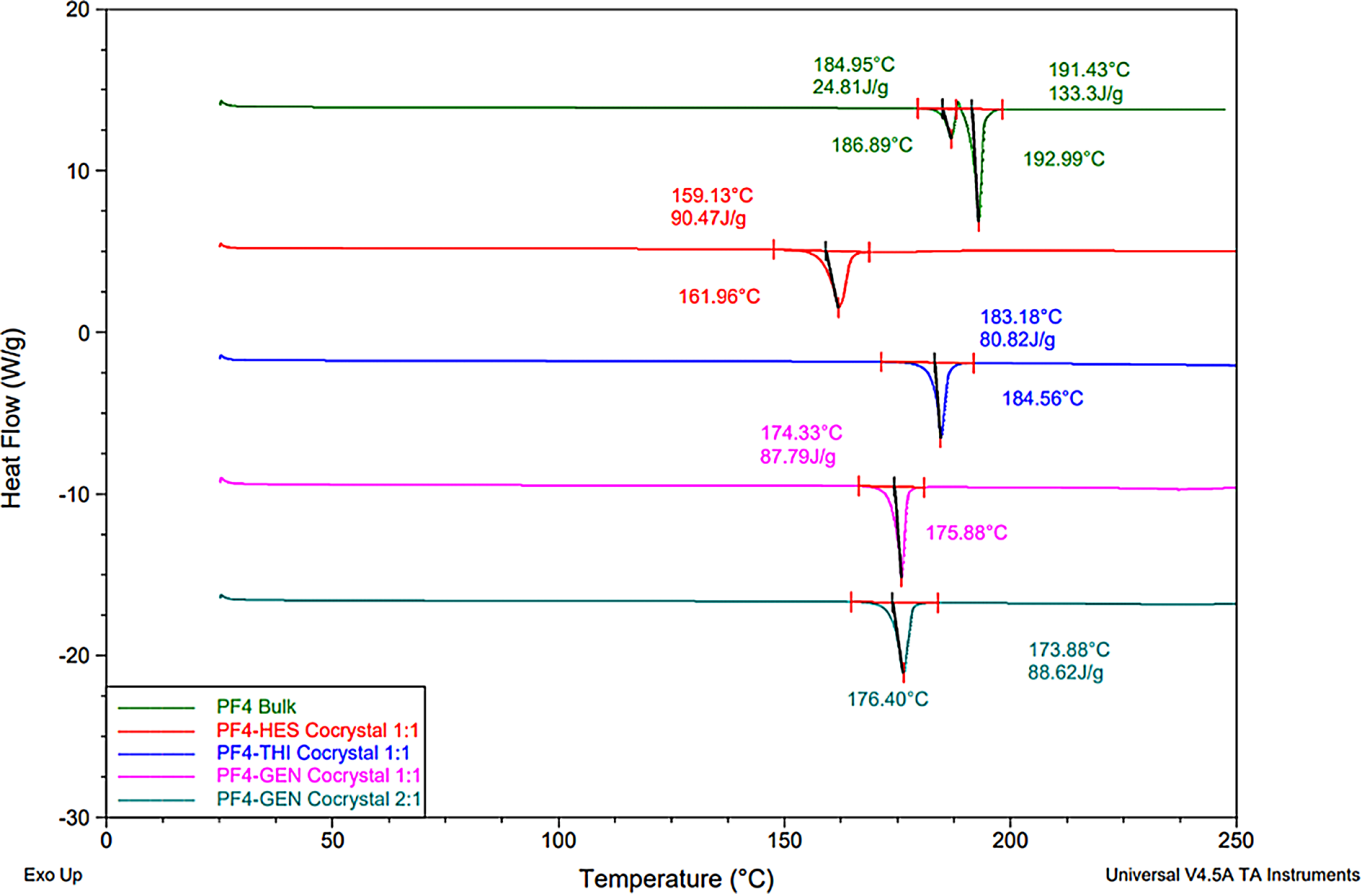
DSC thermograms of bulk PF4 (green) and PF4-HES cocrystal (Red), PF4 Thiabendazole cocrystal (Blue), PF4-GEN 1:1 cocrystal (Magenta) and PF4-GEN Cocrystal 2:1 (Teal) after jet dispensing screening

Of the 42 trials conducted, 8 of the candidates indicated showed evidence of cocrystalization after characterization via DSC. Three cocrystals, with thiabendazole, genistein or hesperitin showed a single endothermic melt, with no melting peak correlating with either of the original cocrystal constituents. Figure 9 shows the DSC thermograms of the successful jet dispensed cocrystal trials overlaid with that of PF4. The DSC thermogram for PF4 exhibits two endothermic events divided by an exotherm. The first endothermic peak is observed with an onset value of 184.95 °C, this is followed by a barely discernible exotherm at 188.19 °C with the second endotherm occurring immediately after with an onset value of 191.43 °C.

This melting temperature was consistent across triplicate batches and a variety of scanning rates (5 °C/sec, 10 °C/sec, 20 °C/sec and 50 °C/sec) carried out to separate any overlapping events, of which there was none. Upon cooling and re-heating, the PF4 samples recrystallize at 174.4 °C and upon reheating the melting points occur on the same position.

The thermograms from the PF4-THI mixture (Fig. 1S, Supplementary Materials) revealed a single melt occurring at around 183.0 °C (± 1.0), for the 1:1 and 1:1.1 samples. This endotherm was unique from that of either melting point of PF4 or THI at 304.3 °C, which is consistent with values reported in literature [34]. The presence of a single, sharp endothermic melt independent of the bulk constituents is indicative of cocrystal formation [1, 2]. The 1:2 stoichiometric sample also displayed a single melting peak, although observed at a higher temperature of 187.5 °C. An additional small shoulder may be indicative of impurities in the melt, with the presence of the coformer impacting post melt behaviour [35]. The 2:1 melting endotherm is near identical to that of the bulk PF4 form, indicating that the increased amount of API has caused either the THI to become miscible within the API or no interaction has occurred between the two materials [36]. This is evident by the fact that only the PF4 remains, while there is no THI residue within the API/coformer mixture. Numerous sharp events are noted within a broad endotherm, indicative of artifacts in the data set created during degradation above 270 °C. As the melting point of THI is above 300 °C, and therefore any melt for this component would be obscured by the degradation onset. The 1:1.1 sample also displayed a slight shoulder when returning to baseline at 187 °C, and the 1:1 sample shows a slightly greater normalized enthalpy change (80.82 J/g in 1:1, compared to 79.1 J/g in 1:1.1). This indicates the new form is near identical, but the 1:1 mixture result in a purer entity, thus the 1:1 binary mixture of PF4/THI was decided to be the optimal form for cocrystallization via HME.

The PF4/GEN mixtures displayed a single endotherm around 174 °C (± 1.0), which is different from the melting points of both PF4 and bulk GEN (306 °C), indicating a new crystal form has crystallized (Fig. 2S). The PF4/GEN cocrystal seems to be the most stable, being able to form across the different array of stoichiometric ratios, though signs of degradation can be seen in the 1:2 sample, indicating that the cocrystal may not be fully formed. As the melting point and enthalpy is nearly identical across all stoichiometric ratios, it can be assumed that the same crystal form has crystallized, despite the difference in ratio.

The 1:1 and 1:1.1 PF4/HES combinations, displayed a single melting point at 158 °C, with no endotherm relating to PF4 or HES present, indicating a new crystal structure has formed. The full results (Fig. 3S, Supplementary Materials) show that the 2:1 sample had a much broader melt, ranging from 145.6 to 166 °C, indicating an impure product, while the 1:2 showed a broad secondary melt ranging from 181 to 205 °C, with the peak max at 200.2 °C. This broad secondary melt in the 1:2 sample can likely be attributed to remaining HES in the sample, that did not interact with the PF4, due to the larger quantity. It should be noted that the endotherm does not immediately return to baseline after the melt, with a peak shoulder being visible from 166 °C to 178 °C. This indicates impurity in the sample.

Of particular interest was the PF4 mixtures with LTY and LTP coformers (Fig. 4, 5 S Supplementary Materials). Both coformers ranked high on the initial complementarity screen and showed a propensity for hetero H-bonding. However, the thermogram for PF4 cocrystals with both LTY and LTP displays a near identical melt profile to that of the pure API, indicating that supramolecular homosynthons have formed between PF4 functional groups, instead of heterosynthons with the coformers. “The reported melting temperature of LTY is at 344°C as this is above the degradation temperature of PF4, the DSC was not heated to this higher temperature, and it is assumed that bulk LTY remains in the mixture and no interaction took place [37]. The LTP mixtures show a broad melting event ranging from 265–295, with multiple events occurring in this range. These events were not further investigated as it falls out of the range of this study, but as the reported melting temperature of LTP is at 290°C, it can be assumed that bulk LTP remains in the mixture and no interaction occurred between it and PF4 [38].

The DSC data seems to indicate that the NIC part of the PF4/NIC mixtures were competing against the PF4 component to form H-bonds with like molecules. All ratios display a sharp endothermic peak in the range 122–126 °C, the enthalpy loss displayed in the endothermic peaks correlates with the amount of NIC in the physical mixture (Fig. 6S, Supplementary Materials). This range is slightly depressed from NICs melting point of 128 °C, indicating an impure melting of the substance. There is a second broad endotherm ranging from around 150–170 °C, which could either be attributed to a depressed PF4 melt or the melting of a new form. Given that the 2:1 and 1:1.1 samples contain a peak at 186 °C and 189 °C respectively indicates that some pure PF4 remains in both mixtures, suggesting that maybe the broad peak occurring between 150 and 170 °C is an impure melt of a new crystal form. Given that the double peak has disappeared and an increase in the API component correlates to a smaller endotherm, indicates some heterosynthon formation has taken place between the NIC and PF4, but the competitive H-bonding from the NIC coformer may have prevented pure cocrystallization [1, 2, 33]. Due to these interesting results, a further two mixtures of PF4 and NIC were jet dispensed in ratios of 3:1 and 4:1 to evaluate if an increased proportion of API would result in less residual NIC and hopefully a new, sharper endothermic peak indicative of cocrystallization. Unfortunately, the residual NIC content remained, and an increased level of residual PF4 was found indicating this API/coformer pair do not form a cocrystal.

A similar trend is seen in the PF4/ETB mixtures, where there is a sharp endothermic peak between 116 and 119 °C, which corresponds with the reported melting point for ETB [39]. All mixtures display broad endotherms indicating the presence of impure melts, most likely involving the residual PF4 (Fig. 7S, Supplementary Materials). Interestingly, for the mixtures with a higher ratio of PF4 (2:1), the endotherm is elevated, while for mixtures involving more coformer, the endotherm is depressed. This indicates a limited interaction between the API and coformer [1, 2]. Another example of limited interaction can be seen on the PF4/PAM mixtures, where the characteristic peaks of PF4 can be seen, with just a reduced peak enthalpy, and a broad, impure melting endotherm can be seen above 200 °C. In the 1:2 mixture, more interaction was seen between the two constituents, with one of the characteristic twin melting points, reduced and the peak at 215 °C, slightly sharpened. A test was performed to scan for residual Pamoic acid above 300 °C (the melt temperature of Pamoic acid), but unfortunately, the melt event was overlapped by degrading PF4 content, indicating that much more un-cocrystallized material remained in the mixture [40].

The PF4/Biotin mixtures showed promising results for the 1:1, 1:1.1 and 1:2 stoichiometric ratios, with a new peak forming at 181.4 °C, approximately 10 °C lower than the larger peak of PF4, with the smaller second peak absent from the thermogram (Fig. 8S, Supplementary Materials). The 2:1 cocrystal presented a much broader melt and a second endotherm at 185.9 °C, close to the initial PF4 bulk endotherm, indicating remaining PF4 is in the mixture. The other 3 ratios presented no remaining PF4, but it is believed that residual BIO remained at ∼ 210 °C, indicated by a broad, but small enthalpy change. Due to the small amount of impure compound, other stoichiometries were tried in addition to attempting to produce a PF4/BIO cocrystal through ball milling. Unfortunately, the residual BIO remained in the mixture after characterization, indicating that forming a stable cocrystal with no remaining traces of the parent compounds is likely to be unfeasible. Due to the relatively expensive price of BIO, it was not possible to forward this for further investigation unless complete transformation could be proven, therefore studies incorporating BIO were not advanced.

**Fig. 10:**
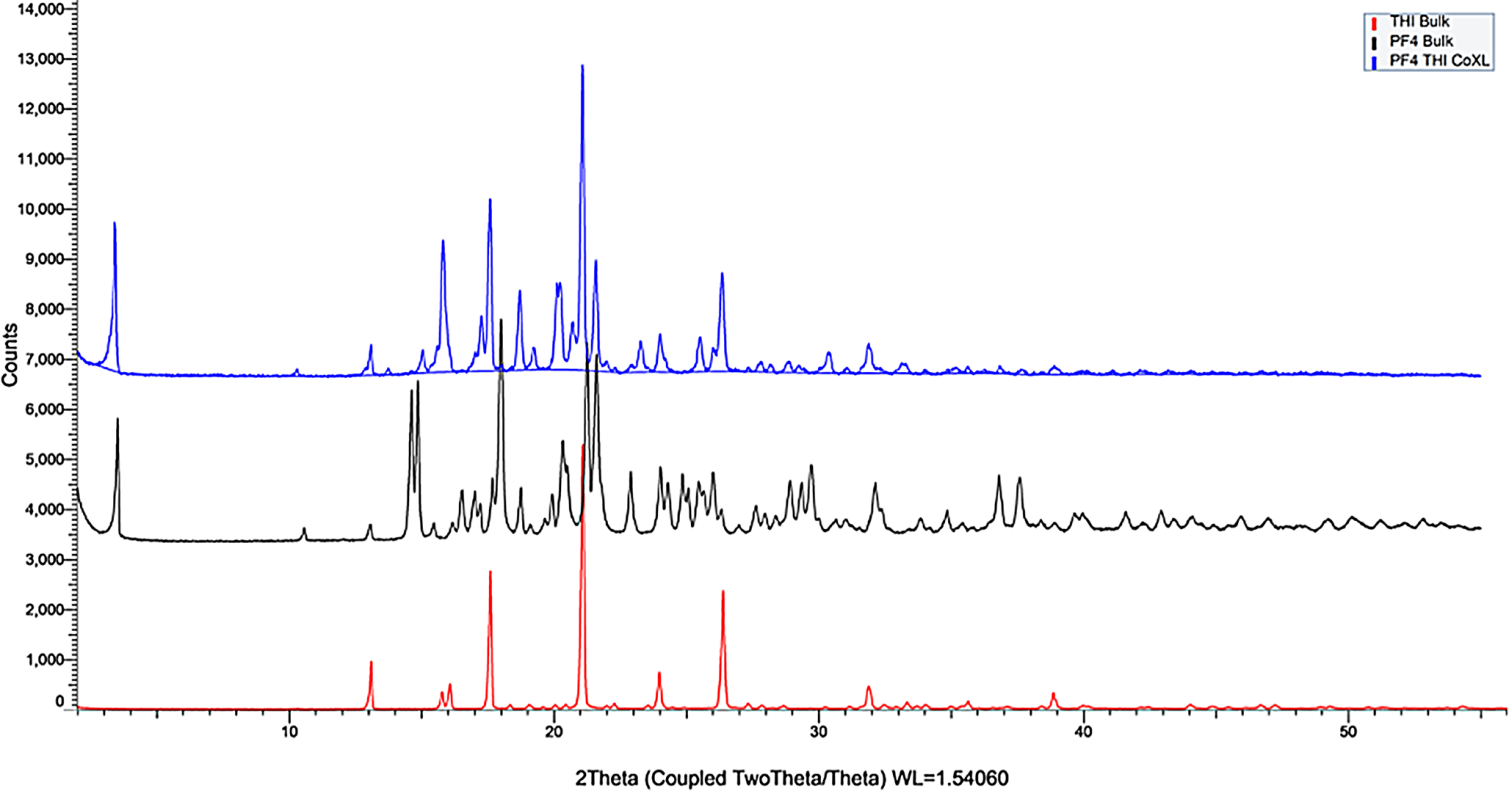
XRPD diffractograms displaying Bulk PF4 (Black), Bulk THI (Red) and the PF4-THI cocrystal (Blue)

To confirm the synthesis of new cocrystals, coformers which showed evidence of cocrystalization with PF4 were again synthesized in larger quantities via jet dispensing, using the same parameters as shown in Table 2. These samples where then characterized via XRPD to confirm the formation of a new chemical entities. As can be seen in Fig. 10, (Figs. 11 and 12, Supplementary Materials), where the three diffractograms for the newly formed structures contain vastly different diffractograms to that of their bulk constituents. Unique intensity peaks of the for the cocrystal with Thiabendazole can be seen at 15.09, 15.80, 17.26, 19.22, 20.11, 23.27 and 30.37 ° 2θ. For Genistine unique peaks, not correlating with the reference structures for parent compounds can be seen at 4.68, 6.79, 10.82, 11.72, 13.65, 16.15, 16.87, 19.42, 29.10 and 30.28 ° 2θ. Lastly, new intensity peaks can be seen at peaks can be observed at 8.97, 11.48, 16.54, 17.42, 19.53, 21.03, 19.94, 22.50 and 26.63 ° 2θ.

## Conclusions

This work demonstrates how a computational screening approach, by analysing trends among successful molecular motifs and the likelihood of hydrogen bond formations, can be combined with HTS jet dispersion screening to produce a QbD method for coformer selection of pharmaceutical cocrystals. Through modelling, it was possible to identify coformer candidate likely to form cocrystals, while high throughput screening allowed the selection of correct stoichiometric ratios, while wasting minimal materials. By using a combined approach of different computational screening methods, an initial short list of 259 potential coformers were narrowed down to 11. After experimental screening, a new cocrystals of PF4 with Hesperitin, Thiabendazole, and Genistein coformers have been characterised. While a success rate of 3 in 11 may seem low, given cocrystallizations unpredictable nature, this method has facilitated the identification of 3 new structures with minimal material usage and manpower. From this assessment, 3 new cocrystal forms will be put forward to scaled-up, continuous manufacture to assess this methods applicability to a commercial production process. Part B of this study will detail the processing of these cocrystals via hot-melt extrusion and fully characterize the new structures to ensure their cocrystallization in a micronized, HTS setting can be replicated on a larger scale to produce a pure batch of PF4 cocrystals.

## Electronic supplementary material

Below is the link to the electronic supplementary material.

Supplementary Material 1

## Data Availability

The data will be provided upon request.
