# Supplementary material for: A quality by design strategy for cocrystal design based on novel computational and experimental screening strategies: part A
Source: Drug Deliv Transl Res. 2024 Nov 20;15(7):2448–66. doi: 10.1007/s13346-024-01743-2 (PMC12137494; doi:10.1007/s13346-024-01743-2)
Supplement: Supplementary file 1 — Supplementary Material 1 [file 13346_2024_1743_MOESM1_ESM.docx]

**Supplementary Information**

**Table 1S:** Mogul geometry check for PF4

| Type | Molecule | Fragment | Classification | No. of hits | Query value | Mean | Std. dev. | z-score | \|x - mean | Minimum | Maximum | Median | \|d(min) | Local density |
| --- | --- | --- | --- | --- | --- | --- | --- | --- | --- | --- | --- | --- | --- | --- |
| bond | PF-04191834 | N2 N1 | Not unusual (enough hits) | 171 | 1.36 | 1.36 | 0.016 | 0.04 | 0.001 | 1.29 | 1.41 | 1.36 | 0 |  |
| bond | PF-04191834 | C3 N1 | Not unusual (enough hits) | 1893 | 1.33 | 1.33 | 0.025 | 0.16 | 0.004 | 1.25 | 1.69 | 1.33 | 0 |  |
| bond | PF-04191834 | C1 N2 | Not unusual (enough hits) | 27 | 1.36 | 1.36 | 0.01 | 0.15 | 0.001 | 1.32 | 1.38 | 1.36 | 0 |  |
| bond | PF-04191834 | C4 N2 | Not unusual (enough hits) | 1618 | 1.46 | 1.46 | 0.018 | 0.03 | 0 | 1.26 | 1.69 | 1.46 | 0 |  |
| bond | PF-04191834 | C2 C1 | Not unusual (enough hits) | 899 | 1.39 | 1.39 | 0.023 | 0.13 | 0.003 | 1.32 | 1.56 | 1.39 | 0 |  |
| bond | PF-04191834 | C5 C1 | Not unusual (enough hits) | 2611 | 1.48 | 1.48 | 0.016 | 0.02 | 0 | 1.30 | 1.59 | 1.48 | 0 |  |
| bond | PF-04191834 | C2 C3 | Not unusual (enough hits) | 278 | 1.39 | 1.39 | 0.019 | 0.04 | 0.001 | 1.26 | 1.48 | 1.39 | 0 |  |
| bond | PF-04191834 | C6 C5 | Not unusual (enough hits) | 10000 | 1.39 | 1.39 | 0.017 | 0.07 | 0.001 | 0.90 | 1.63 | 1.39 | 0 |  |
| bond | PF-04191834 | C10 C5 | Not unusual (enough hits) | 10000 | 1.39 | 1.39 | 0.017 | 0.07 | 0.001 | 0.90 | 1.63 | 1.39 | 0 |  |
| bond | PF-04191834 | C7 C6 | Not unusual (enough hits) | 10000 | 1.38 | 1.38 | 0.018 | 0 | 0 | 0.88 | 1.65 | 1.38 | 0 |  |
| bond | PF-04191834 | C7 C8 | Not unusual (enough hits) | 10000 | 1.39 | 1.39 | 0.02 | 0.04 | 0.001 | 1.09 | 1.69 | 1.39 | 0 |  |
| bond | PF-04191834 | C9 C8 | Not unusual (enough hits) | 10000 | 1.39 | 1.39 | 0.02 | 0.04 | 0.001 | 1.09 | 1.69 | 1.39 | 0 |  |
| bond | PF-04191834 | C8 S1 | Not unusual (enough hits) | 2214 | 1.77 | 1.77 | 0.02 | 0.03 | 0.001 | 1.39 | 1.98 | 1.77 | 0 |  |
| bond | PF-04191834 | C10 C9 | Not unusual (enough hits) | 10000 | 1.38 | 1.38 | 0.018 | 0.001 | 0 | 0.88 | 1.65 | 1.38 | 0 |  |
| bond | PF-04191834 | C11 S1 | Not unusual (enough hits) | 2214 | 1.77 | 1.77 | 0.02 | 0.03 | 0.001 | 1.39 | 1.98 | 1.77 | 0 |  |
| bond | PF-04191834 | C12 C11 | Not unusual (enough hits) | 10000 | 1.39 | 1.39 | 0.02 | 0.01 | 0 | 1.09 | 1.69 | 1.39 | 0 |  |
| bond | PF-04191834 | C16 C11 | Not unusual (enough hits) | 1681 | 1.39 | 1.39 | 0.019 | 0.02 | 0 | 1.21 | 1.60 | 1.39 | 0 |  |
| bond | PF-04191834 | C13 C12 | Not unusual (enough hits) | 10000 | 1.38 | 1.39 | 0.021 | 0.04 | 0.001 | 0.76 | 1.69 | 1.39 | 0 |  |
| bond | PF-04191834 | C13 C14 | Not unusual (enough hits) | 10000 | 1.38 | 1.39 | 0.021 | 0.003 | 0 | 0.76 | 1.69 | 1.39 | 0 |  |
| bond | PF-04191834 | C14 C15 | Not unusual (enough hits) | 10000 | 1.39 | 1.39 | 0.016 | 0.12 | 0.002 | 1.11 | 1.63 | 1.39 | 0 |  |
| bond | PF-04191834 | C16 C15 | Not unusual (enough hits) | 10000 | 1.39 | 1.39 | 0.017 | 0.07 | 0.001 | 1.12 | 1.61 | 1.39 | 0 |  |
| bond | PF-04191834 | C17 C15 | Not unusual (enough hits) | 276 | 1.53 | 1.53 | 0.016 | 0.021 | 0 | 1.44 | 1.62 | 1.53 | 0 |  |
| bond | PF-04191834 | C18 C17 | Not unusual (enough hits) | 144 | 1.53 | 1.55 | 0.014 | 0.94 | 0.013 | 1.50 | 1.64 | 1.55 | 0 |  |
| bond | PF-04191834 | C21 C17 | Not unusual (enough hits) | 144 | 1.53 | 1.55 | 0.014 | 1.07 | 0.015 | 1.50 | 1.64 | 1.55 | 0.001 |  |
| bond | PF-04191834 | C17 C22 | Not unusual (enough hits) | 16 | 1.55 | 1.54 | 0.015 | 0.62 | 0.009 | 1.49 | 1.56 | 1.54 | 0 |  |
| bond | PF-04191834 | C18 C19 | Not unusual (enough hits) | 272 | 1.51 | 1.51 | 0.029 | 0.06 | 0.002 | 1.21 | 1.56 | 1.51 | 0 |  |
| bond | PF-04191834 | O1 C19 | Not unusual (enough hits) | 10000 | 1.44 | 1.42 | 0.041 | 0.56 | 0.023 | 0.68 | 1.95 | 1.42 | 0 |  |
| bond | PF-04191834 | O1 C20 | Not unusual (enough hits) | 10000 | 1.43 | 1.42 | 0.041 | 0.09 | 0.003 | 0.68 | 1.95 | 1.42 | 0 |  |
| bond | PF-04191834 | C21 C20 | Not unusual (enough hits) | 272 | 1.51 | 1.51 | 0.029 | 0.18 | 0.005 | 1.21 | 1.56 | 1.51 | 0 |  |
| bond | PF-04191834 | O2 C22 | Not unusual (enough hits) | 425 | 1.23 | 1.23 | 0.021 | 0.12 | 0.002 | 1.15 | 1.36 | 1.23 | 0 |  |
| bond | PF-04191834 | C22 N3 | Not unusual (enough hits) | 425 | 1.323 | 1.32 | 0.02 | 0.13 | 0.003 | 1.23 | 1.41 | 1.32 | 0 |  |
| angle | PF-04191834 | C3 N1 N2 | Not unusual (enough hits) | 27 | 104.2 | 104.4 | 0.489 | 0.56 | 0.276 | 103.7 | 105.5 | 104.4 | 0.049 |  |
| angle | PF-04191834 | C4 N2 N1 | Not unusual (enough hits) | 171 | 117.1 | 119.1 | 2.189 | 0.94 | 2.056 | 114.9 | 141.8 | 119.1 | 0.057 |  |
| angle | PF-04191834 | C4 N2 C1 | Not unusual (enough hits) | 27 | 127.7 | 128.3 | 4.783 | 0.14 | 0.681 | 104.8 | 130.9 | 129.1 | 0.02 |  |
| angle | PF-04191834 | C2 C1 N2 | Not unusual (enough hits) | 15 | 105.8 | 106.1 | 0.467 | 0.63 | 0.295 | 105.2 | 107.1 | 106.1 | 0.035 |  |
| angle | PF-04191834 | C5 C1 N2 | Not unusual (enough hits) | 63 | 126.2 | 125.2 | 1.366 | 0.72 | 0.98 | 122.5 | 129.1 | 125.2 | 0.155 |  |
| angle | PF-04191834 | C5 C1 C2 | Not unusual (enough hits) | 232 | 127.9 | 127.8 | 1.846 | 0.10 | 0.192 | 122.4 | 132.7 | 127.8 | 0.006 |  |
| angle | PF-04191834 | C3 C2 C1 | Not unusual (enough hits) | 88 | 104.5 | 104.9 | 1.102 | 0.43 | 0.471 | 102.2 | 108.5 | 105.1 | 0.076 |  |
| angle | PF-04191834 | C2 C3 N1 | Not unusual (enough hits) | 223 | 111.986 | 112.4 | 1.381 | 0.29 | 0.406 | 102.9 | 117.3 | 112.4 | 0.003 |  |
| angle | PF-04191834 | C6 C5 C1 | Not unusual (enough hits) | 5043 | 120.9 | 120.7 | 1.923 | 0.11 | 0.206 | 101.6 | 138.6 | 120.7 | 0 |  |
| angle | PF-04191834 | C10 C5 C1 | Not unusual (enough hits) | 5043 | 120.6 | 120.7 | 1.923 | 0.06 | 0.118 | 101.6 | 138.6 | 120.7 | 0 |  |
| angle | PF-04191834 | C10 C5 C6 | Not unusual (enough hits) | 10000 | 118.5 | 118.6 | 1.425 | 0.03 | 0.036 | 56.1 | 134.7 | 118.6 | 0 |  |
| angle | PF-04191834 | C7 C6 C5 | Not unusual (enough hits) | 10000 | 120.7 | 120.8 | 1.163 | 0.03 | 0.032 | 89.8 | 133.3 | 120.8 | 0 |  |
| angle | PF-04191834 | C6 C7 C8 | Not unusual (enough hits) | 8079 | 120.4 | 120.5 | 1.204 | 0.07 | 0.086 | 105.9 | 133.1 | 120.4 | 0 |  |
| angle | PF-04191834 | C9 C8 C7 | Not unusual (enough hits) | 9620 | 119.1 | 118.8 | 1.676 | 0.15 | 0.252 | 92.1 | 135.6 | 118.9 | 0.001 |  |
| angle | PF-04191834 | C7 C8 S1 | Not unusual (enough hits) | 4043 | 120.5 | 120.3 | 3.06 | 0.04 | 0.132 | 102.9 | 137.7 | 120.3 | 0.001 |  |
| angle | PF-04191834 | C9 C8 S1 | Not unusual (enough hits) | 4043 | 120.5 | 120.7 | 3.06 | 0.01 | 0.126 | 102.9 | 137.7 | 120.3 | 0.001 |  |
| angle | PF-04191834 | C10 C9 C8 | Not unusual (enough hits) | 8079 | 120.4 | 120.5 | 1.204 | 0.08 | 0.09 | 105.9 | 133.1 | 120.4 | 0 |  |
| angle | PF-04191834 | C9 C10 C5 | Not unusual (enough hits) | 10000 | 120.8 | 120.8 | 1.163 | 0.01 | 0.016 | 89.8 | 133.3 | 120.8 | 0 |  |
| angle | PF-04191834 | C11 S1 C8 | Not unusual (enough hits) | 508 | 103. | 103.1 | 2.143 | 0.02 | 0.032 | 96.434 | 109.9 | 103.2 | 0.007 |  |
| angle | PF-04191834 | C12 C11 S1 | Not unusual (enough hits) | 4043 | 120.4 | 120.3 | 3.06 | 0.02 | 0.064 | 102.9 | 137.7 | 120.3 | 0 |  |
| angle | PF-04191834 | C16 C11 S1 | Not unusual (enough hits) | 358 | 119.8 | 119.8 | 2.955 | 0.009 | 0.026 | 111.6 | 127.3 | 119.5 | 0.001 |  |
| angle | PF-04191834 | C12 C11 C16 | Not unusual (enough hits) | 662 | 119.8 | 119.8 | 1.404 | 0.02 | 0.032 | 111.1 | 133.1 | 119.8 | 0.003 |  |
| angle | PF-04191834 | C13 C12 C11 | Not unusual (enough hits) | 8861 | 119.7 | 120.0 | 1.714 | 0.20 | 0.346 | 87.4 | 156.8 | 120.0 | 0 |  |
| angle | PF-04191834 | C14 C13 C12 | Not unusual (enough hits) | 10000 | 120.1 | 120.2 | 1.419 | 0.11 | 0.155 | 70.6 | 141.4 | 120.2 | 0 |  |
| angle | PF-04191834 | C13 C14 C15 | Not unusual (enough hits) | 10000 | 121.0 | 120.8 | 1.05 | 0.27 | 0.278 | 101.8 | 132.5 | 120.8 | 0 |  |
| angle | PF-04191834 | C14 C15 C16 | Not unusual (enough hits) | 10000 | 118.3 | 117.8 | 1.93 | 0.29 | 0.566 | 104.3 | 130.8 | 117.3 | 0 |  |
| angle | PF-04191834 | C14 C15 C17 | Not unusual (enough hits) | 537 | 121.5 | 121.0 | 1.889 | 0.26 | 0.484 | 114.7 | 126.5 | 121.0 | 0.013 |  |
| angle | PF-04191834 | C16 C15 C17 | Not unusual (enough hits) | 614 | 120.1 | 121.2 | 1.89 | 0.58 | 1.102 | 115.1 | 126.8 | 121.3 | 0.001 |  |
| angle | PF-04191834 | C11 C16 C15 | Not unusual (enough hits) | 16 | 120.9 | 120.9 | 1.179 | 0.01 | 0.083 | 119.1 | 124.1 | 120.8 | 0.003 |  |
| angle | PF-04191834 | C18 C17 C15 | Not unusual (enough hits) | 68 | 108.4 | 111.5 | 2.073 | 1.49 | 3.082 | 106.8 | 116.4 | 111.6 | 0.229 |  |
| angle | PF-04191834 | C15 C17 C22 | Not unusual (enough hits) | 20 | 102.8 | 107.0 | 2.897 | 1.44 | 4.161 | 101.3 | 111.9 | 106.4 | 1.111 |  |
| angle | PF-04191834 | C21 C17 C18 | Not unusual (enough hits) | 33 | 109.3 | 108.0 | 1.095 | 1.40 | 1.533 | 105.5 | 110.5 | 107.8 | 0.055 |  |
| angle | PF-04191834 | C18 C17 C22 | Not unusual (enough hits) | 16 | 114.4 | 108.3 | 3.338 | 1.82 | 6.088 | 102.2 | 111.7 | 109.9 | 2.716 |  |
| angle | PF-04191834 | C21 C17 C22 | Not unusual (enough hits) | 16 | 114.9 | 108.3 | 3.338 | 1.97 | 6.586 | 102.2 | 111.7 | 109.9 | 3.214 |  |
| angle | PF-04191834 | C19 C18 C17 | Not unusual (enough hits) | 15 | 110.6 | 112.0 | 1.102 | 1.40 | 1.541 | 109.2 | 113.1 | 112.4 | 0.085 |  |
| angle | PF-04191834 | O1 C19 C18 | Not unusual (enough hits) | 134 | 111.4 | 111.6 | 1.057 | 0.13 | 0.141 | 108.6 | 117.9 | 111.5 | 0.003 |  |
| angle | PF-04191834 | C20 O1 C19 | Not unusual (enough hits) | 8737 | 110.4 | 109.9 | 3.339 | 0.15 | 0.501 | 63.5 | 161.0 | 109.8 | 0 |  |
| angle | PF-04191834 | O1 C20 C21 | Not unusual (enough hits) | 134 | 111.9 | 111.6 | 1.057 | 0.29 | 0.302 | 108.6 | 117.9 | 111.5 | 0.005 |  |
| angle | PF-04191834 | C20 C21 C17 | Not unusual (enough hits) | 15 | 111.0 | 112.1 | 1.102 | 0.95 | 1.05 | 109.2 | 113.1 | 112.4 | 0.048 |  |
| angle | PF-04191834 | O2 C22 C17 | Not unusual (enough hits) | 29 | 121.1 | 120.9 | 1.063 | 0.19 | 0.202 | 118.0 | 122.5 | 121.193 | 0.007 |  |
| angle | PF-04191834 | C17 C22 N3 | Not unusual (enough hits) | 29 | 116.0 | 116.9 | 1.173 | 0.70 | 0.823 | 115.3 | 120.0 | 116.7 | 0.018 |  |
| angle | PF-04191834 | O2 C22 N3 | Not unusual (enough hits) | 425 | 122.8 | 123.2 | 1.999 | 0.16 | 0.322 | 103.9 | 129.9 | 123.2 | 0.003 |  |
| angle | PF-04191834 | C1 N2 N1 | Unusual (enough hits) | 15 | 110.4 | 112.2 | 0.555 | 3.24 | 1.8 | 111.4 | 113.3 | 112.2 | 0.985 |  |
| angle | PF-04191834 | C21 C17 C15 | Unusual (enough hits) | 68 | 106.2 | 111.5 | 2.073 | 2.54 | 5.255 | 106.8 | 116.4 | 111.6 | 0.535 |  |
| torsion | PF-04191834 | C6 C5 C1 N2 | Not unusual (enough hits) | 1154 | 44.9 |  |  |  |  |  |  |  | 0.007 | 0.29 |
| torsion | PF-04191834 | C10 C5 C1 N2 | Not unusual (enough hits) | 1154 | 135.1 |  |  |  |  |  |  |  | 0 | 0.28 |
| torsion | PF-04191834 | C6 C5 C1 C2 | Not unusual (enough hits) | 3033 | 135.0 |  |  |  |  |  |  |  | 0.017 | 0.29 |
| torsion | PF-04191834 | C10 C5 C1 C2 | Not unusual (enough hits) | 3033 | 44.9 |  |  |  |  |  |  |  | 0.029 | 0.29 |
| torsion | PF-04191834 | C7 C8 S1 C11 | Not unusual (enough hits) | 3830 | 0.001 |  |  |  |  |  |  |  | 0.001 | 0.06 |
| torsion | PF-04191834 | C9 C8 S1 C11 | Not unusual (enough hits) | 3830 | -179.9 |  |  |  |  |  |  |  | 0.02 | 0.07 |
| torsion | PF-04191834 | C12 C11 S1 C8 | Not unusual (enough hits) | 3830 | 83.0 |  |  |  |  |  |  |  | 0.01 | 0.09 |
| torsion | PF-04191834 | C16 C11 S1 C8 | Not unusual (enough hits) | 3830 | -96.8 |  |  |  |  |  |  |  | 0.02 | 0.09 |
| torsion | PF-04191834 | C18 C17 C15 C14 | Not unusual (enough hits) | 216 | -12.4 |  |  |  |  |  |  |  | 0.516 | 0.14 |
| torsion | PF-04191834 | C21 C17 C15 C14 | Not unusual (enough hits) | 216 | -129.8 |  |  |  |  |  |  |  | 0.01 | 0.11 |
| torsion | PF-04191834 | C22 C17 C15 C14 | Not unusual (enough hits) | 45 | 109.1 |  |  |  |  |  |  |  | 2.881 | 0.07 |
| torsion | PF-04191834 | C18 C17 C15 C16 | Not unusual (enough hits) | 216 | 168.3 |  |  |  |  |  |  |  | 0.118 | 0.17 |
| torsion | PF-04191834 | C21 C17 C15 C16 | Not unusual (enough hits) | 216 | 50.8 |  |  |  |  |  |  |  | 0.154 | 0.14 |
| torsion | PF-04191834 | O2 C22 C17 C15 | Not unusual (enough hits) | 40 | 131.8 |  |  |  |  |  |  |  | 0.435 | 0.23 |
| torsion | PF-04191834 | C15 C17 C22 N3 | Not unusual (enough hits) | 38 | -47.5 |  |  |  |  |  |  |  | 0.998 | 0.21 |
| torsion | PF-04191834 | O2 C22 C17 C18 | Not unusual (enough hits) | 40 | -110.9 |  |  |  |  |  |  |  | 0.267 | 0.15 |
| torsion | PF-04191834 | C18 C17 C22 N3 | Not unusual (enough hits) | 40 | 69.9 |  |  |  |  |  |  |  | 1.274 | 0.18 |
| torsion | PF-04191834 | O2 C22 C17 C21 | Not unusual (enough hits) | 40 | 16.9 |  |  |  |  |  |  |  | 0.774 | 0.28 |
| torsion | PF-04191834 | C21 C17 C22 N3 | Not unusual (enough hits) | 40 | -162.4 |  |  |  |  |  |  |  | 1.026 | 0.28 |
| torsion | PF-04191834 | C22 C17 C15 C16 | Unusual (enough hits) | 45 | -70.2 |  |  |  |  |  |  |  | 11.068 | 0 |
| ring | PF-04191834 | C5 C6 C7 C8 C9 C10 | Not unusual (enough hits) | 500 |  |  |  |  |  | 0.197 | 12.0 |  |  | 0.99 |
| ring | PF-04191834 | C11 C12 C13 C14 C15 C16 | Not unusual (enough hits) | 500 |  |  |  |  |  | 0.1 | 14.2 |  |  | 0.99 |
| ring | PF-04191834 | C17 C18 C19 O1 C20 C21 | Not unusual (enough hits) | 18 |  |  |  |  |  | 0.25 | 35.9 |  |  | 0.94 |
| ring | PF-04191834 | N1 N2 C1 C2 C3 | Unusual (enough hits) | 70 |  |  |  |  |  | 10.2 | 13.1 |  |  | 0 |

**Table 2S:**  Original ranked order of coformer screening list before manual refinement. List is firstly ranked by the amount of API conformations for which the complementarity screening was a success and secondly by the optimal deviation from Lazlo’s preferred parameters. Results in green indicate the conformer passed the complementarity screen, H-bond prop screen and has a good solubility range. Purple indicates the coformer passed the complementarity screen but were revealed to have poor H bonding ability after the HBP screening. Results in yellow were predicted to be likely to form a supramolecular heterosynthon but failed the complementarity screen on 1 or more conformations. Results in orange indicate that some crystal packing space group data was incomplete and was manually filled in. This, however, made it impossible to generate interaction maps, excluding them from this section of the screening. Results in red indicate the coformer was not screened beyond the initial complementarity rankings detailed in section 6.3.2. This could be due to either early disqualification as a result of Disorder, incomplete crystal data, Mercury 4.2 being unable to generate the structural data or the structure being a solvate or salt. Coformers in white which lie below the cut-off point if rank 60 were not screened beyond the initial complementarity screening simply due to too low on the complementarity rankings to warrant further investigation.

**Fig 1S:** Overlaid DSC thermograms for PF4/THI cocrystal mixtures in stoichiometric

ratios of 1:1 (Blue), 1:1.1 (Green), 2:1 (Red) and 1:2 (Brown).

**Fig 2S:** Overlaid DSC thermograms for PF4/GEN cocrystal mixtures in stoichiometric

ratios of 1:1 (Blue), 1:1.1 (Green), 2:1 (Red) and 1:2 (Brown).

**Fig. 3S:** Overlaid DSC thermograms for PF4/HES cocrystal mixtures in stoichiometric ratios of 1:1 (Blue), 1:1.1 (Green), 2:1 (Red) and 1:2 (Brown).

**Fig. 4S:** Overlaid DSC thermograms for PF4/LTY cocrystal mixtures in stoichiometric ratios of 1:1 (Blue), 1:1.1 (Green), 2:1 (Red) and 1:2 (Brown).

**Fig. 45:** Overlaid DSC thermograms for PF4/LTP cocrystal mixtures in stoichiometric ratios of 1:1 (Blue), 1:1.1 (Green), 2:1 (Red) and 1:2 (Brown).

**Fig. 6S:** Overlaid DSC thermograms for PF4/NIC cocrystal mixtures in stoichiometric ratios of 1:1 (Blue), 1:1.1 (Green), 2:1 (Red) and 1:2 (Brown).

**Fig 7S:** Overlaid DSC thermograms for PF4/ETB cocrystal mixtures in stoichiometric ratios of 1:1 (Blue), 1:1.1 (Green), 2:1 (Red) and 1:2 (Brown).

**Fig. 8S:** Overlaid DSC thermograms for PF4/BIO cocrystal mixtures in stoichiometric ratios of 1:1 (Blue), 1:1.1 (Green), 2:1 (Red) and 1:2 (Brown)
